# Nonsurgical management of hallux valgus: findings of a randomised pilot and feasibility trial

**DOI:** 10.1186/s13047-023-00677-1

**Published:** 2023-11-13

**Authors:** Hylton B. Menz, Polly Q. X. Lim, Sheree E. Hurn, Karen J. Mickle, Andrew K. Buldt, Matthew P. Cotchett, Edward Roddy, Anita E. Wluka, Bircan Erbas, Mehak Batra, Shannon E. Munteanu

**Affiliations:** 1 School of Allied Health, Human Services and Sport, La Trobe University, Melbourne, VIC 3086 Australia; 2 School of Clinical Sciences, Faculty of Health, Queensland University of Technology, Kelvin Grove, QLD 4059 Australia; 3 Applied Sport Science and Exercise Testing Laboratory, College of Health, Medicine and Wellbeing, University of Newcastle, Ourimbah, NSW 2258 Australia; 4 Primary Care Centre Versus Arthritis, School of Medicine, Keele University, Keele, Staffordshire, ST5 5BG UK; 5 Haywood Academic Rheumatology Centre, Midlands Partnership University NHS Foundation Trust, Haywood Hospital, Burslem, Staffordshire, ST6 7AG UK; 6 School of Public Health and Preventive Medicine, Monash University, 553 St Kilda Rd, Melbourne, VIC 3004 Australia; 7 School of Psychology and Public Health, La Trobe University, Melbourne, VIC 3086 Australia

## Abstract

**Background:**

Hallux valgus is a common and disabling condition. This randomised pilot and feasibility trial aimed to determine the feasibility of conducting a parallel group randomised trial to evaluate the effectiveness of a nonsurgical intervention for reducing pain associated with hallux valgus.

**Methods:**

Twenty-eight community-dwelling women with painful hallux valgus were randomised to receive either a multifaceted, nonsurgical intervention (footwear, foot orthoses, foot exercises, advice, and self-management) or usual care (advice and self-management alone). Outcome measures were obtained at baseline, 4, 8 and 12 weeks. The primary outcome was feasibility, evaluated according to demand (recruitment rate and conversion rate), acceptability, adherence, adverse events, and retention rate. Limited efficacy testing was conducted on secondary outcome measures including foot pain, foot muscle strength, general health-related quality of life, use of cointerventions, and participants’ perception of overall treatment effect.

**Results:**

Between July 8, 2021, and April 22, 2022, we recruited and tested 28 participants (aged 44 to 80 years, mean 60.7, standard deviation 10.7). This period encompassed two COVID-related stay-at-home orders (July 16 to July 27, and August 5 to October 21, 2021). The predetermined feasibility thresholds were met for retention rate, foot pain, mental health-related quality of life, and use of cointerventions, partly met for acceptability, adverse events, and muscle strength, and not met for demand (recruitment rate or conversion rate), adherence, physical health-related quality of life and perception of overall treatment effect.

**Conclusion:**

In its current form, a randomised trial of footwear, foot orthoses, foot exercises, advice and self-management for relieving pain associated with hallux valgus is not feasible, particularly due to the low adherence with the intervention. However, it is difficult to determine whether the trial would be feasible under different circumstances, particularly due to COVID-19 stay-at-home orders. Future trials will need to consider improving the aesthetics of the footwear and making the exercise program less burdensome.

**Trial registration:**

Australian and New Zealand Clinical Trial Registry (ACTRN12621000645853).

**Supplementary Information:**

The online version contains supplementary material available at 10.1186/s13047-023-00677-1.

## Background

Hallux valgus is characterised by the lateral deviation of the hallux towards the lesser toes which disrupts the alignment of the first metatarsophalangeal joint. A systematic review reported pooled prevalence estimates of 23% in people aged 18 to 65 years and 36% in people aged over 65 years [1], with women twice as likely to be affected than men. The subluxation of the first metatarsophalangeal joint and formation of an osseus prominence often leads to abnormal gait patterns [2], impaired balance [3], difficulties with finding comfortable footwear [4], increased risk of falls [5] and decreased health-related quality of life [6]. Although many cases require surgery [7], there is considerable interest as to whether a nonsurgical approach is effective [8].

Nonsurgical management of hallux valgus involves footwear advice or modification, foot orthoses, night splints, and physical therapies. In clinical practice, these interventions are often combined in a multifaceted approach [9]. However, there is limited evidence for the effectiveness of these interventions, when used either alone or in combination. A recent systematic review and meta-analysis of nonsurgical interventions for hallux valgus identified 16 parallel-group and crossover studies evaluating a wide range of nonsurgical interventions, and only one evaluated a multifaceted approach [8]. Overall, included trials were of low methodological quality and many had small sample sizes and short follow-up periods, thus providing low certainty as to the effectiveness of these interventions and the longer-term management of the condition.

The most commonly used nonsurgical treatments are footwear, foot orthoses and foot exercises [9]. Ill-fitting footwear is a potentially important modifiable risk factor, as population-based case-control studies have suggested that the likelihood of having the condition is greater in those who have worn shoes with a narrow toe box in their twenties [10], and that wearing narrow shoes may lead to the initial development of the condition [11]. Foot orthoses have been shown to reduce load under the hallux and midfoot in people with hallux valgus [12], and a randomised controlled trial reported a reduction in pain at 6 months in those who received this intervention [13]. A recent trial in older people has also found that a progressive, resistance exercise program improved hallux plantarflexion strength by approximately 20% over 12 weeks [14], which may be beneficial in the treatment of hallux valgus [15].

The evidence provides support for the use of footwear, foot orthoses and foot exercises for the treatment of hallux valgus, but these approaches are yet to be evaluated in combination. Therefore, the primary objective of this study was to evaluate the feasibility of conducting a randomised trial comparing multifaceted, nonsurgical intervention versus usual care for reducing pain associated with hallux valgus. The secondary objectives were to provide a signal of efficacy to justify a future trial and obtain statistical parameters to inform the main trial sample size calculation.

## Methods

The full study protocol has already been published [16], and key components are reproduced here.

### Study design

The multifaceted intervention for hallux valgus (MARVELL) trial was a parallel group, participant- and assessor-blinded, randomised pilot and feasibility trial over 12 weeks [17]. The study was registered with the Australian and New Zealand Clinical Trial Registry (ACTRN12621000645853), and the protocol was developed in consultation with the Standard Protocol Items: Recommendations for Interventional Trials (SPIRIT) 2013 statement [18], the CONSORT 2010 statement extension to randomised pilot and feasibility trials [19], and items from CONSORT, as recommended by Thabane and Lancaster [20]. Ethical approval was obtained from the La Trobe University Human Ethics Committee (reference number: HEC20474).

### Participants

Twenty-eight participants were recruited via from the northern suburbs of Melbourne, Victoria, Australia via postal invitation using a database of patients who received podiatry treatment at the La Trobe University Health Sciences Clinic, email distribution to staff members in the School of Allied Health, Human Services and Sport at La Trobe University, Facebook advertising and posters placed in the local community. To be eligible for inclusion, participants: (i) were aged ≥ 40 years, (ii) were female, (iii) had pain in the big toe joint/s (i.e. first metatarsophalangeal or interphalangeal) for at least 12 weeks, (iv) had big toe joint pain rated at least 3 out of 10 on a numerical rating scale, (v) were able to walk household distances (more than 50 m) unaided, (vi) were capable of understanding the English language, and (vii) had at least moderate hallux valgus on one or both feet [21]. Participants were not eligible for inclusion if they self-reported: (i) surgical treatment for hallux valgus on either foot, (ii) lower limb or partial foot amputation, (iii) an inflammatory rheumatological condition or connective tissue disease, (iv) a neurological disease which interfered with walking, (v) having worn arch-contouring foot orthoses in the past 12 weeks, (vi) having performed foot exercises in the past 12 weeks, or (vii) an injury of lower limb(s) or back that may interfere with reaching their feet.

### Sample size

This was a pilot and feasibility trial, so was not fully powered to detect statistically significant differences between the groups. The recommended sample size for pilot studies is 12 people per group [22], however to allow for a 15% drop-out rate, we recruited 28 participants.

### Recruitment and screening

Participants were recruited using postal invitation using a database of patients who had recently received podiatry treatment at the La Trobe University Health Sciences Clinic, email distribution to staff members in the School of Allied Health, Human Services and Sport at La Trobe University, Facebook advertising and posters placed in the local community. Potential participants were asked to contact the chief investigator (HBM) to express their interest and were then screened for eligibility by either of two members of the research team (HBM and PQXL).

### Baseline assessments

Participant characteristics were collected by structured interview at the baseline assessment and included age, height, weight, country of birth, education level, major medical conditions, and medications. The following questionnaires and clinical assessments were also conducted: the Manchester scale for hallux valgus [21], foot pain characteristics [23], shoe-wearing history [10], the Incidental and Planned Activity Questionnaire [24] and the Credibility/Expectancy Questionnaire [25].

### Randomisation

Permuted block randomisation (with block sizes of four, six and eight) was used to randomise participants on a 1:1 ratio to the control or intervention group using an online randomisation service (www.sealedenvelope.com).

### Study procedure

All face-to-face assessments were performed in the Foot and Ankle Laboratory at La Trobe University, Victoria, Australia. Postal follow-ups were conducted at 4 and 8 weeks, with the final face-to-face follow-up at 12 weeks.

### Blinding

Participants were blinded to group allocation by limited disclosure, in that they were told that the clinical trial was comparing two nonsurgical treatments for hallux valgus, but they were not informed about the specific characteristics of the treatments. Research staff administering the treatments could not be blinded. Outcomes were participant-reported, thus this study was also assessor-blinded (as participants were blinded). The study biostatistician performing the statistical analyses (BE) was blinded.

### Interventions

#### Control group

The control group received a self-management package based on United Kingdom National Health Service recommendations [26] which advise people with hallux valgus to wear wide shoes with a low heel and soft sole, apply cold-packs and silicone gel bunion pads, and use paracetamol for pain relief. We provided all participants with cold-packs (Hot + Cold Therapy Gel Pack; OAPL, Clayton, Victoria, Australia) and silicone gel bunion pads (Spandex Gel™ Cushion Bunion Pads; Neat^®^ Feat, Auckland, New Zealand). To meet ethical guidelines and aid retention, on completion of the study the control group participants were offered the same treatment as the intervention group.

#### Intervention group

**Fig. 1:**
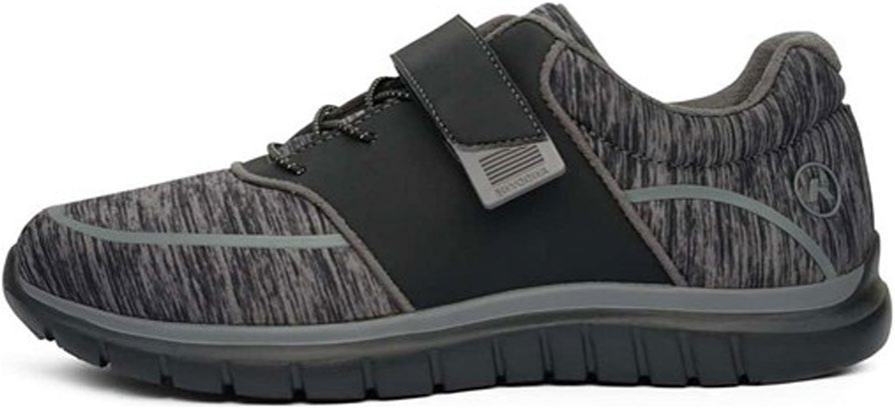
Intervention footwear (Anodyne #45 Sport Jogger; Global Footcare, Coomera, Queensland, Australia). Reproduced from *J Foot Ankle Res* 2022;15:45

**Fig. 2:**
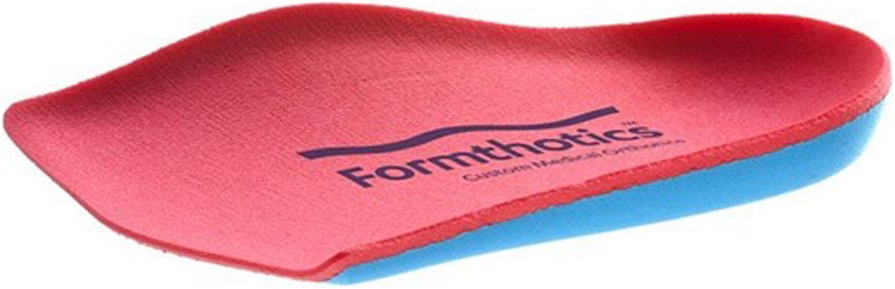
Intervention foot orthoses (dual-density, three-quarter length Formthotics™). Image reproduced with permission from Foot Science International, Christchurch, New Zealand. Reproduced from *J Foot Ankle Res* 2022;15:45

The intervention group were provided with the same advice and self-management package as the control group, in addition to:

(i) Footwear: high quality, off-the-shelf footwear (Anodyne #45 Sport Jogger; Global Footcare, Coomera, Queensland, Australia) (Fig. 1).
(ii) Foot orthoses: prefabricated Formthotics™ (Foot Science International, Christchurch, New Zealand). These orthoses were three-quarter length and were constructed from dual-density, closed-cell polyethylene foam (Fig. 2). We used the three-quarter length as they are less likely to increase dorsal/medial pressure from footwear compared to the full-length devices [27], and used a heat-gun to warm the devices prior to fitting. No custom modifications were added.
(iii) Foot exercises: participants were provided with access to a smart-phone app (PhysiTrack^®^, London, United Kingdom) which demonstrated a home-based version of the progressive resistance foot exercise program developed by Mickle et al. [14]. The set of 14 exercises (including four ‘warm-up’ and two ‘cool-down’ exercises) were performed three times per week for the 12 weeks. Each session took approximately 30 min to complete. Participants were contacted by the developer of the exercise program (KJM) to address any queries and ensure they were performing the exercises correctly. See Supplementary file for the list of exercises.

Interventions were administered to both feet. Participants were free to use additional treatments during the study if they were documented in the 4-weekly postal surveys. However, participants were withdrawn from the study if they reported undergoing surgical intervention.

### Primary outcome

The primary outcome was feasibility, which was evaluated according to demand, acceptability, adherence, adverse events and retention rate [28]. *Demand* was determined by the recruitment rate (participants recruited per month) and the conversion rate (participants providing consent divided by those who met the selection criteria). The recruitment rate was considered acceptable if six eligible participants were recruited per month, and the conversion rate was considered acceptable if ≥ 75% of those who were eligible participated. *Acceptability* of the intervention was determined using questions from the Monitor Orthopaedic Shoes (MOS) questionnaire [29] which addressed issues such as appearance, comfort, weight, and ease of donning and doffing. The intervention was considered acceptable if ≥ 75% of the intervention group scored more than 5/10 for each of questions 1–6. *Adherence* to the footwear/orthoses intervention was documented using 4-weekly diaries and objectively assessed over 12 weeks using a small temperature sensor embedded in the orthosis (Orthotimer^®^, Balingen, Germany) [30]. Adherence was considered acceptable if ≥ 75% of participants wore the footwear/orthoses for an average of ≥ 5 h per day over the 12-week follow-up period. Adherence to the exercise program was documented using 4-weekly diaries (or the PhysiTrack^®^ smart-phone app) and was considered acceptable if ≥ 75% of participants attempted at least 66% of the total number of exercise sessions (i.e., 24 out of 36 sessions). In both the control and intervention groups, adherence to the hot/cold packs and bunion pads were measured using 4-weekly diaries. *Adverse events* were assessed at 4-weekly intervals via postal diary. Serious adverse events were defined as events that were life-threatening, required hospitalisation, or resulted in persistent or significant disability or incapacity [31]. The rate of adverse events was considered acceptable if < 15% and none were considered serious. *Retention rate* was the proportion of recruited participants who completed the 12-week outcome assessment. A ≥ 80% retention rate in each group was considered acceptable.

### Secondary outcome

One of the secondary objectives was limited efficacy testing of the outcome measures. The key secondary outcome measure was the pain subscale of the Manchester-Oxford Foot Questionnaire (MOXFQ) [32, 33]. The minimum clinically important difference for the MOXFQ pain subscale is 12 points [34].

Other limited efficacy outcome measures included the MOXFQ [32] walking/standing and social subscales, measured at baseline and at 4-weekly intervals until 12 weeks, foot and ankle muscle strength, measured with a hand-held dynamometer using our previously documented, reliable protocol at baseline and week 12 [35], general health-related quality of life, assessed using Short Form (SF) 12 [36] measured at 4-weekly intervals, number of participants using co-interventions, documented at 4-weekly intervals, and participants’ perception of overall treatment effect, assessed with the question “Overall, how has your foot pain changed since the start of the study?” and using a global impression of change 15-point Likert scale response (ranging from ‘a very great deal worse’ to ‘a very great deal better’), measured at 12 weeks [37].

An acceptable feasibility outcome for the limited efficacy testing was a signal of efficacy for each continuously-scored outcome measure, as evidenced by at least a small effect size (Cohen’s *d* ≥ 0.20, calculated as the difference between the two group means divided by the overall standard deviation), less than 20% use of cointerventions in the intervention group, and a greater than 25% difference in the proportion of participants reporting at least ‘somewhat better’ on the perception of overall treatment effect compared to the control group.

### Statistical analysis

As this was a pilot and feasibility study, it was not powered to detect changes in outcome measures, so the focus was not on inferential testing (although this was conducted) [17]. Descriptive statistics were used to report feasibility outcomes. Mean (SD) scores and mean differences (95% CI) were used to explore differences in continuous variables between the groups. Differences in the MOXFQ pain subscale between groups at 12 weeks (analysis of covariance, adjusted for baseline differences) were used to inform the sample size calculation for the main randomised trial.

## Results

### Participant characteristics

**Fig. 3:**
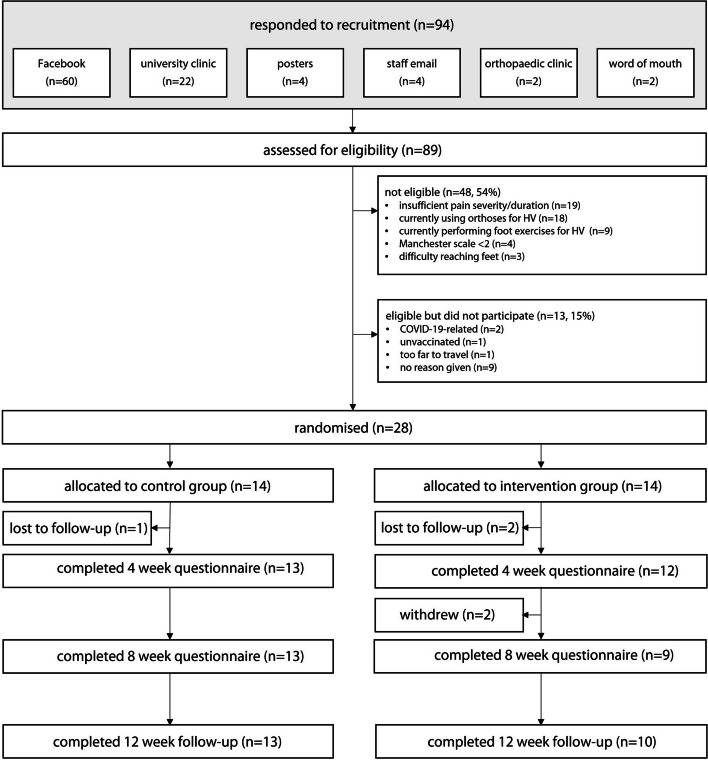
Participant flow through the trial

Between July 8, 2021, and April 22, 2022, we recruited 89 potential participants for eligibility and then randomised and tested 28 participants (aged 44 to 80 years, mean 60.7, standard deviation 10.7). This period encompassed two COVID-related stay at home orders (July 16 to July 27 and August 5 to October 21, 2021). Forty-eight people who responded were not eligible, and 13 were eligible but chose not to participate. During the study, three participants were lost to follow-up (one in the control group and two in the intervention group) and two withdrew; one was unwell, and one had to look after a family member with COVID-19 (both were in the intervention group). This led to 13 and 10 participants completing the 12 week follow-up in the control and intervention groups, respectively. Characteristics of participants are shown in Table 1, and a flow-chart of participants throughout the trial is shown in Fig. 3.

**Table 1:** Participant characteristics. Values are mean (SD) unless otherwise stated

Characteristic	Control group (*n* = 14)	Intervention group (*n* = 14)	Total sample (*n* = 28)
Age, years	63.6 (10.7)	57.9 (10.3)	60.7 (10.7)
Height, cm	160.0 (8.2)	160.2 (5.6)	160.1 (6.9)
Weight, kg	69.0 (13.4)	70.5 (9.3)	69.8 (11.3)
Body mass index, kg/m^2^	26.9 (4.7)	27.5 (3.8)	27.2 (4.2)
Education level, n (%)			
Primary	0 (0)	1 (7.1)	1 (3.6)
Secondary	6 (42.9)	2 (14.3)	8 (28.6)
Tertiary	3 (21.4)	4 (28.6)	7 (25.0)
College / university / postgraduate	5 (35.7)	7 (50.0)	12 (43.0)
Ethnicity, n (%)			
Oceanian^a^	7 (50.0)	6 (42.9)	13 (46.4)
North African	1 (7.1)	3 (42.0)	4 (14.3)
Anglo-Indian	2 (14.3)	0 (0)	2 (7.1)
South-East Asian	2 (14.3)	0 (0)	2 (7.1)
Southern and Central Asian	1 (7.1)	1 (7.1)	2 (7.1)
Southern and Eastern European	1 (7.1)	1 (7.1)	2 (7.1)
Sub-Saharan African	0 (0)	1 (7.1)	1 (3.6)
North-West European	0 (0)	1 (7.1)	1 (3.6)
United Kingdom	0 (0)	1 (7.1)	1 (3.6)
Medical conditions, n (%)			
Hypertension	7 (50.0)	2 (14.3)	9 (32.1)
Osteoarthritis	5 (35.7)	4 (28.6)	9 (32.1)
Heart disease	1 (7.1)	2 (14.3)	3 (10.7)
Cancer	2 (14.3)	0 (0)	2 (7.1)
Leg ulcers	1 (7.1)	0 (0)	1 (3.6)
Use of ≥ 4 medications	3 (21.4)	3 (21.4)	6 (21.4)
General health			
SF12 physical	42.0 (10.8)	47.6 (10.9)	44.8 (11.0)
SF12 mental	50.7 (9.6)	46.7 (8.0)	48.7 (8.9)
Incidental physical activity, total hours / week	46.9 (18.3)	33.7 (22.5)	40.3 (21.2)
Foot pain			
MOXFQ pain	56.1 (21.2)	53.9 (20.4)	55.0 (20.5)
MOXFQ walking/standing	37.2 (26.6)	43.9 (30.3)	40.6 (28.2)
MOXFQ social	42.4 (27.7)	40.2 (23.9)	41.3 (25.4)
MOXFQ total	44.4 (21.7)	46.1 (23.7)	45.3 (22.4)
Pain elsewhere in the foot, n (%)			18 (64.3)
Hallux valgus, n (%)			
Side affected (both / left / right)	12 (85.7) / 0 (0) / 2 (14.3)	11 (78.6) / 1 (7.1) / 2 (14.3)	23 (82.1) / 1 (3.6) / 4 (14.3)
Index foot (right / left)	9 (64.3) / 5 (35.7)	9 (64.3) / 5 (35.7)	18 (64.3) / 10 (35.7)
Moderate (Manchester scale = 2)	10 (71.4)	9 (64.3)	19 (67.9)
Severe (Manchester scale = 3)	4 (28.6)	5 (35.7)	9 (32.1)
Previous or current treatment, n (%)			
Changed footwear	8 (57.1)	10 (71.4)	18 (64.3)
Insoles from pharmacy	5 (35.7)	4 (28.6)	9 (32.1)
Foot exercises	4 (28.6)	5 (35.7)	9 (32.1)
Bunion pads	4 (28.6)	2 (14.3)	6 (21.4)
Credibility and expectancy of intervention^b^			
Credibility	26.0 (22.9)	21.4 (3.9)	22.8 (3.2)
Expectancy	19.4 (4.0)	19.4 (4.1)	19.4 (4.0)

*SF12* Short Form 12 Health Survey, *MOXFQ* Manchester-Oxford Foot Questionnaire

^a^All participants identified as Australian

^b^Higher scores indicate greater credibility and expectancy. Highest score for each is 27

### Primary outcome

Table 2 provides a summary of the results for the feasibility outcome measures. Demand, as determined by the recruitment rate and the conversion rate (the proportion of participants providing consent of those who met the selection criteria) was not met (2.8 participants and 68%, respectively). Acceptability of the intervention, as determined using questions from the MOS questionnaire [29] was partly met, as while only 28% considered the footwear to be attractive to others and only 50% considered the footwear to be attractive to themselves, questions 4, 5 and 6 (relating to fit, ease of use and weight) met the criteria. Adherence to the footwear/orthoses intervention, as documented using 4-weekly diaries and objectively assessed over 12 weeks using the Orthotimer^®^, was not met using either method (57% and 14%, respectively) nor was adherence to the exercise program (documented using 4-weekly diaries; 34% or the PhysiTrack^®^ smart-phone app; 7%). Adverse events were common (64%) but not serious, so were considered partly met, and overall retention was high (82%) and met the criteria.

**Table 2:** Summary of feasibility outcome measures and thresholds

Feasibility construct	Outcome measure	Predetermined threshold	Result	Achieved?
Demand	Recruitment rate	6 participants per month	2.8	No
	Conversion rate	≥ 75%	68%	No
Acceptability	MOS questionnaire	≥ 75% of the intervention group score more than 5/10 for each of questions 1–6	28–93%	Partly^a^
Adherence				
Footwear/orthoses	Orthotimer^®^ sensor	≥ 75% of participants wear the footwear/orthoses for an average of ≥ 5 h per day	14%	No
	4-weekly diaries		57%	No
Exercise	PhysiTrack^®^ app	≥ 75% of participants complete at least 24/36 (66%) of the exercise sessions	7%	No
	4-weekly diaries		43%	No
Adverse events	4-weekly diaries	< 15% and no serious events	64%	Partly^b^
Retention rate	Proportion of participants followed up at 12 weeks	≥ 80% retention	82%	Yes

^a^Only 28% thought the footwear was attractive to others, and only 50% judged it be attractive to themselves. Questions 4, 5 and 6 (relating to fit, ease of use and weight) met the criteria

^b^Although 64% of the sample reported developing pain elsewhere, none were considered serious events and all resolved during the trial

### Secondary outcome

**Fig. 4:**
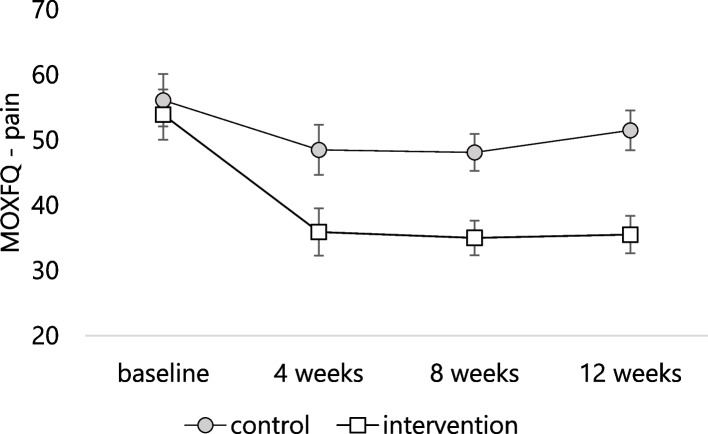
Manchester-Oxford Foot Questionnaire pain score in intervention and control groups during the 12 weeks of the study. Error bars are standard errors

Table 3 provides a summary of results for the pilot and feasibility outcome measures and predetermined thresholds, Tables 4 and 5 provide MOXFQ, SF12 measures and strength measures, respectively, at baseline and follow-up, and Fig. 4 shows the improvement in the MOXFQ pain subscale. Table 4 shows that after adjusting for baseline values, a statistically significant adjusted mean difference of -9.5 (95% CI -0.8 to -18.2, effect size *d* = 0.55) in the MOXFQ pain score was found in favour of the intervention group. Although participants in the intervention group improved in all other MOXFQ domains at 12 weeks, none of these were statistically significant. None of the SF12 change scores were statistically significant.

**Table 3:** Summary of the limited efficacy outcome measures

Efficacy measure	Predetermined threshold	Result	Achieved?
MOXFQ pain	small effect size (*d* ≥ 0.20)	0.55	Yes
MOXFQ walking/standing	small effect size (*d* ≥ 0.20)	0.14	No
MOXFQ social	small effect size (*d* ≥ 0.20)	0.04	No
MOXFQ total	small effect size (*d* ≥ 0.20)	0.25	Yes
Muscle strength	small effect size (*d* ≥ 0.20)	0.14–0.71	Partly^a^
SF12 physical	small effect size (*d* ≥ 0.20)	0.14	No
SF12 mental	small effect size (*d* ≥ 0.20)	0.28	Yes
Use of cointerventions	< 20% in intervention group	14%	Yes
Perception of overall treatment effect	≥ 25% difference between groups in proportion reporting at least “somewhat better”	5%	No

*MOXFQ* Manchester-Oxford Foot Questionnaire, *SF12* Short Form 12 Health Survey

^a^Did not meet threshold for lesser toe plantarflexion *(d* = 0.14)

**Table 4:** Efficacy outcome measures at baseline and follow-up. Values are mean (SD), *P*=p value, *d*=effect size

	Control group (*n* = 14)	Intervention group (*n* = 14)	Adjusted mean difference (95% CI)^a^	*P*	*d*
MOXFQ pain^b^					
Baseline	56.1(21.2)	53.9 (20.4)			
4 weeks	48.5 (20.3)	35.9 (19.2)			
8 weeks	48.1 (14.9)	35.0 (14.1)			
12 weeks	51.5 (16.1)	35.5 (15.2)	-9.5 (-0.8 to -18.2)	0.035	0.55
MOXFQ standing^b^					
Baseline	38.2 (27.4)	36.4 (30.3)			
4 weeks	32.8 (27.0)	25.0 (19.0)			
8 weeks	26.2 (19.7)	31.7 (23.7)			
12 weeks	31.3 (21.5)	27.1 (21.1)	-3.0 (-11.9 to 5.8)	0.487	0.14
MOXFQ social^b^					
Baseline	42.8 (28.7)	33.8 (24.5)			
4 weeks	38.0 (26.4)	27.5 (18.2)			
8 weeks	26.6 (25.6)	26.4 (15.6)			
12 weeks	32.2 (21.6)	25.6 (20.7)	-0.8 (-12.2 to 10.5)	0.880	0.04
MOXFQ total^b^					
Baseline	44.4 (21.7)	46.1 (23.7)			
4 weeks	39.1 (21.7)	32.3 (21.2)			
8 weeks	33.4 (16.2)	28.3 (19.2)			
12 weeks	37.9 (16.3)	29.4 (18.3)	-4.2 (-11.2 to 2.8)	0.215	0.25
SF12 physical^c^					
Baseline	42.1 (10.8)	47.6 (10.9)			
4 weeks	44.1 (8.4)	48.0 (10.1)			
8 weeks	46.0 (9.2)	49.9 (5.1)			
12 weeks	43.9 (11.8)	48.8 (6.8)	1.5 (-6.9 to 9.8)	0.720	0.14
SF12 mental^c^					
Baseline	50.7 (9.6)	46.7 (8.0)			
4 weeks	50.8 (8.4)	47.4 (5.9)			
8 weeks	50.6 (8.2)	49.1 (10.4)			
12 weeks	50.7 (11.2)	50.9 (10.4)	3.1 (-4.2 to 10.3)	0.386	0.28

*MOXFQ* Manchester-Oxford Foot Questionnaire, *SF12* Short Form 12 Health Survey

^a^Adjusted for baseline score using analysis of covariance

^b^Higher scores indicate worse symptoms

^c^Higher scores indicate better symptoms/function

**Table 5:** Efficacy strength measures at baseline and follow-up. Values are mean (SD) Newtons, *P*=p value, *d*=effect size

	Control group (*n* = 14)	Intervention group (*n* = 14)	Adjusted mean difference (95% CI)^a^	*P*	*d*
Ankle dorsiflexion					
Baseline	108.0 (29.4)	98.6 (28.9)			
12 weeks	111.8 (31.4)	127.5 (23.7)	20.6 (-1.3 to 42.5)	0.064	0.71
Ankle plantarflexion					
Baseline	111.9 (26.9)	99.5 (25.7)			
12 weeks	125.5 (33.9)	137.9 (30.2)	19.7 (-9.4 to 48.8)	0.172	0.61
Ankle inversion					
Baseline	83.7 (29.8)	69.6 (16.9)			
12 weeks	93.8 (33.9)	94.6 (19.5)	15.3 (-3.4 to 33.9)	0.105	0.56
Ankle eversion					
Baseline	62.3 (24.3)	62.7 (17.0)			
12 weeks	80.3 (24.4)	92.0 (15.3)	12.9 (-2.2 to 27.9)	0.089	0.61
Lesser toe plantarflexion					
Baseline	40.0 (13.8)	35.2 (12.8)			
12 weeks	40.0 (13.1)	40.4 (8.5)	1.6 (-8.3 to 11.5)	0.741	0.14
Hallux plantarflexion					
Baseline	42.2 (10.7)	37.4 (11.5)			
12 weeks	42.9 (17.8)	55.1 (18.8)	12.9 (-2.7 to 28.6)	0.101	0.68

^a^Adjusted for baseline score using analysis of covariance

Mean score values in the intervention and the control groups for strength measures are displayed in Table 5. On average, participants in the intervention group exhibited greater ankle dorsiflexion and eversion strength compared to those in the control group at 12 weeks, although both findings were borderline statistically significant. Improvements were observed in other strength measures but none of these were statistically significant.

The predetermined limited efficacy threshold, based on detecting an effect size of at least 0.20, was met for the MOXFQ pain and total subscales, partly met for muscle strength (this outcome did not meet threshold for lesser toe plantarflexion but met the threshold for all other measurements) and met for SF12 mental (but not SF12 physical). The predetermined thresholds were met for use of cointerventions (14.3% of the intervention group reported use of cointerventions compared to 35.7% of the control group) but not met for perception of overall treatment effect (41.7% of the intervention group reported feeling at least ‘somewhat better’ compared to 36.4% of the control group, a difference between groups of 5%).

### Adherence to other components of the intervention

The cold packs were used from 0 to 56 days (mean 12.1, SD 15.6) during the 12 weeks of the study. Reasons for not using them were “I didn’t need to” (stated on 26 occasions), “bunions don’t hurt during the day” (stated on three occasions), and “cannot tolerate cold” (three occasions), “shoes were comfortable enough” (two occasions), “did not think of using them” (one occasion), “didn’t find any difference in pain” (one occasion) and “limited time” (one occasion). The bunion pads were worn 0 to 552 h (mean 146.4, SD 192.8) during the 12 weeks of the study. Common reasons for not wearing them were “no need” (stated on 22 occasions), “uncomfortable” (10 occasions), “too loose” (four occasions), “too large” (three occasions), and “I lost them” (one occasion).

### Sample size for main trial

Our final secondary objective was to obtain statistical parameters to inform the main trial sample size calculation. We calculated that a fully powered parallel-group superiority trial would require 122 participants (i.e.: 61 per group) using a previously-determined minimal clinically important difference in the efficacy outcome measure (MOXFQ pain subscale) of 12 points [34], standard deviation of 20.4 (recorded in the intervention group of this pilot and feasibility trial), alpha of 5% and power of 90%.

## Discussion

The primary objective of this study was to evaluate the feasibility of conducting a randomised trial comparing a multifaceted nonsurgical intervention versus usual care for reducing pain associated with hallux valgus. This is the first study designed to explore the effects of these interventions when used in combination [9]. We found that the predetermined feasibility thresholds were met for retention rate, the MOXFQ pain and total subscales, SF12 mental, and use of cointerventions, partly met for acceptability, adverse events, and muscle strength, and not met for recruitment rate, conversion rate, adherence, SF12 physical health and perception of overall treatment effect. We also found that our limited disclosure method seems to maintain blinding, as credibility and expectancy were quite high, and there was no difference between the groups. Based on our findings, however, we consider a fully powered randomised trial is not feasible in its current form, particularly due to the low adherence associated with the intervention.

Before discussing the findings in detail, it is worth outlining how COVID-19 may have affected the conduct of the trial. The first participant completed baseline testing on July 8, 2021, however, due to stay-at-home restrictions, recruitment and data collection were suspended from July 15 to 27 and from August 5 to October 21, 2021 (94 days in total) [38], as participants were unable to travel during this time. This also resulted in most of the trial being conducted at a time where physical activity was markedly reduced [39], so the low levels of adherence with the footwear and orthoses – interventions that are designed to be worn outdoors – are perhaps not surprising. Adherence was also very low for the exercise component, which may reflect the impact of stay-at-home restrictions on mental health, particularly in women [39]. Although it is difficult to attribute these low levels of adherence to COVID-19 (and it is possible that similarly low levels of adherence could have been reported under ‘normal’ conditions), our adherence is much lower than previous trials we have conducted involving footwear and/or orthoses [40–44]. However, despite not meeting many of our feasibility thresholds, we did witness a signal of efficacy, which suggests that a future trial could be feasible if modifications could be made to improve demand and adherence.

In designing the original protocol for this study, we acknowledged that the most likely barrier to acceptability of the intervention would be aesthetic concerns regarding the footwear, as the shoes required an extra wide and deep toe box [16]. Indeed, acceptability was only partly met, with 28% considering the footwear to be attractive to others and 50% considering the footwear to be attractive to themselves. Interestingly, two-thirds of the sample had previously tried changing their footwear as a treatment for hallux valgus. The limited efficacy measures were met for the pain and total MOXFQ subscales and the SF12 mental scale, suggesting that those who did adhere to the intervention received some benefit, particularly in relation to foot pain. Although our sample is too small to identify those most likely to benefit, anecdotal evidence suggests that older participants were more satisfied with the footwear and therefore more likely to wear them. This is consistent with previous findings, in that women are more likely to wear shoes with a broader toe box as they age [10], and that older people with foot pain are generally prepared to wear shoes with a broad, wide toe box [41]. If a future trial is planned, it is possible that adherence to the footwear component would be higher in an older cohort and that the intervention could therefore potentially be effective in this age-group. Alternatively, sourcing a shoe that does not compress the toe region but is more aesthetically pleasing may be worthwhile.

Adherence was even lower for the exercise component of the study, with only 7% of the sample completing at least 75% of the exercise sessions according to the PhysiTrack^®^ app. Although this was associated with trends towards improvements in muscle strength, it is unlikely that this level of adherence is acceptable for a fully powered trial, and it is far lower than what is generally accepted as sufficient [45]. In a previous study, Mickle et al. [14] reported very high adherence (89%) with this program, although this involved face-to-face group exercise classes. Group exercise is more effective than home based exercises for hallux valgus [46], but this was not feasible in the current study and is difficult to maintain beyond a trial [47]. Participants found the exercise program quite challenging, and although many were housebound for much of the study due to COVID-19-related stay-at-home restrictions, they generally found it difficult to incorporate the program into their daily routine. In the future, a scaled-down exercise program may be feasible, focusing specifically on the muscle groups known to be affected by hallux valgus (i.e.: those responsible for hallux plantarflexion and abduction) [15].

Interestingly, we found very little association between objectively measured and self-reported adherence. For footwear and orthoses, we found that only 14% wore the devices for an average of ≥ 5 h per day (measured using a temperature sensor) [30], whereas the self-completion diaries indicated a much higher level of adherence (57%). Similarly, we found that only 7% were considered adherent to exercise using the PhysiTrack^®^ app, whereas the self-completion diaries indicated adherence of 43%. This observation is consistent with Nicolson et al. [48], who found that exercise was overestimated in self-completion diaries compared to accelerometers concealed in an ankle cuff weights in people with knee osteoarthritis. These findings suggest that due to the risk of over-reporting, caution needs to be taken when depending on diaries to document adherence.

The findings of this study need to be interpreted in the context of its strengths and limitations. The study design had several key strengths, including randomisation, concealed allocation, and blinded analysis, and the reporting adheres to both the SPIRIT 2013 statement [18] and the CONSORT 2010 statement extension to randomised pilot and feasibility trials [19]. We selected intervention components that have demonstrated safety and acceptability and were relatively low cost and accessible in the Australian context, and selected outcome measures that would provide insights into potential mechanisms of effectiveness. However, several limitations need to be acknowledged. First, as previously mentioned, this trial was conducted during COVID-19, so it is difficult to know whether the low adherence we witnessed was due to stay-at-home restrictions or the nature of the intervention itself. Second, this was a pilot and feasibility trial, so it was not adequately powered to evaluate efficacy. Third, we only assessed women, but we acknowledge that men also develop hallux valgus [11] and that our findings may differ in men.

## Conclusion

This pilot and feasibility study has shown that a fully powered randomised trial of footwear, foot orthoses, foot exercises, advice and self-management for relieving pain associated with hallux valgus is not feasible in its current form due to the low adherence associated with the intervention. However, it is difficult to determine whether the trial would be feasible under different circumstances, as a signal of efficacy was observed, and the trial was conducted during two COVID-19 stay-at-home orders. Future trials may indeed be feasible if adherence to the footwear and exercise interventions can be improved, the aesthetics of the footwear can be enhanced, and the exercise program can be made simpler and less burdensome for participants to undertake.

### Supplementary Information

**Additional file 1.** Description of exercises – see weeks for sets, repetitions and progression.

## Data Availability

De-identified data may be accessed by reasonable request from the corresponding author.

## Abbreviations

CONSORT: Consolidated Standards of Reporting Trials
MOS: Monitor Orthopaedic Shoes
MOXFQ: Manchester-Oxford Foot Questionnaire
SF: Short Form
SPIRIT: Standard Protocol Items: Recommendations for Interventional Trials
