# Supplementary material for: Nonsurgical management of hallux valgus: findings of a randomised pilot and feasibility trial
Source: J Foot Ankle Res. 2023 Nov 13;16:78. doi: 10.1186/s13047-023-00677-1 (PMC10642001; doi:10.1186/s13047-023-00677-1)
Supplement: Supplementary file 1 — Additional file 1. Description of exercises – see weeks for sets, repetitions and progression. [file 13047_2023_677_MOESM1_ESM.pdf]

Description of exercises – see weeks for sets, repetitions and progression

HV Foot strengthening

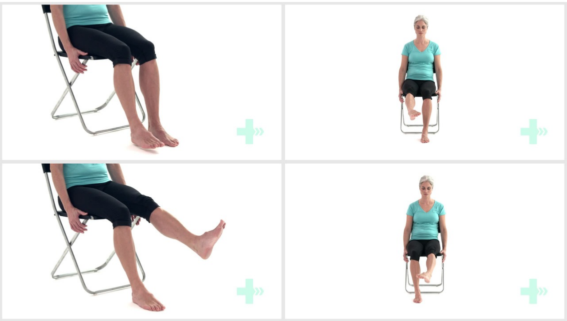

1. Ankle circles seated

The ankle circles help with flexibility of your ankles, this is important for walking and balance.
Sit upright with your back supported before straightening your leg to do the ankle circles.
Lift your leg up and begin making large slow circles with your foot in one direction for the required number of repetitions.
Switch legs and make circles with the other ankle in the same direction.
Switch legs again and reverse the direction of the movement, perform the required number of repetitions firstly with one ankle and then repeat with the other.
Try to stretch your ankle as much as you can as you feel the stretch in your ankle, foot, and in your lower leg.

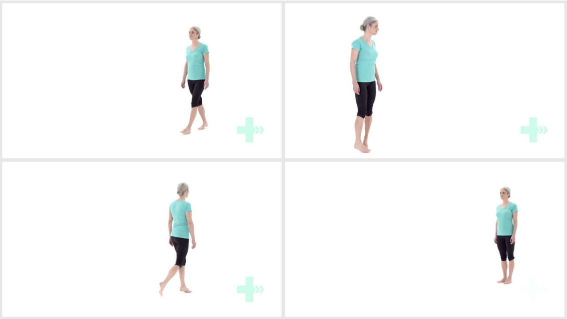

2. Toe walking

Start up on your toes with your heels raised off the floor.
Walk forward with a natural arm swing, keeping your heels off the floor.

Do not do if painful or feeling unbalanced. Alternate between toe and heel walking sets

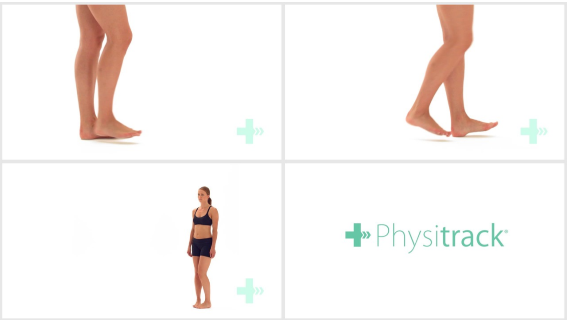

3. Walking on heels

Start from a neutral standing position.
Walk forward balancing only on your heels.

Do not do if you have any heel pain or feel unbalanced

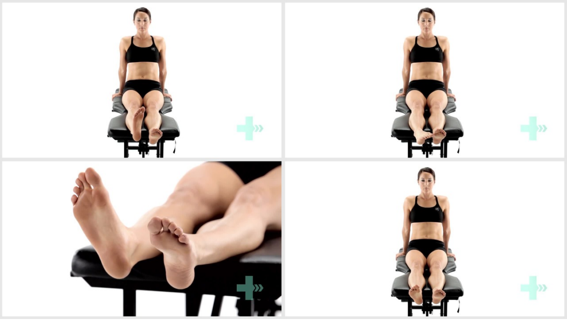

4. Alphabet exercise - ankle AROM

Sit down with your legs extended.
Draw the capital letters of the alphabet with your ankle, accentuating all of the ranges of motion.

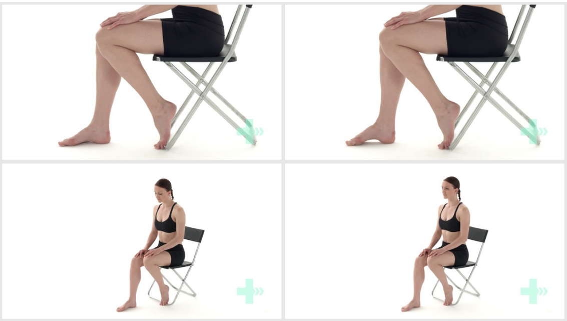

5. Metatarsal doming

Sit up straight in a chair.
Place your foot flat on the floor.
Keeping your toes straight, tighten the muscles through the sole of your foot.
Your arch should raise a little.
Ensure you do not curl your toes under.
You should be able to see your toenails throughout this exercise.
Hold this position.

Hold the position for 5 seconds then relax and repeat for the assigned number of repetitions before switching to the other foot.

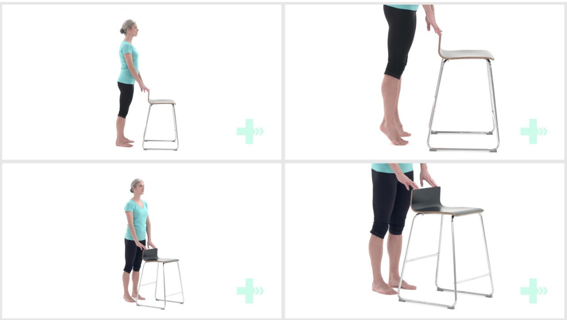

6. Calf raise holding on

Stand with both legs straight and at hip-width apart.
Hold on to a wall or supportive object for balance.
Rise up onto your toes, keeping your legs straight, and control the movement back down to the start position.

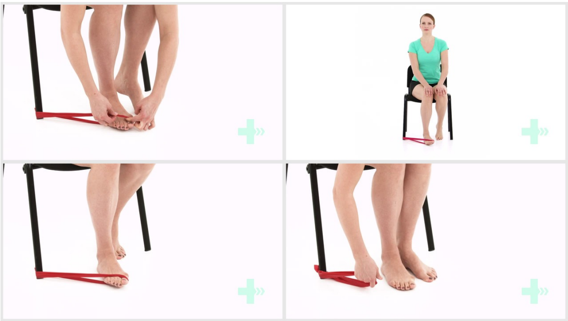

7. Resisted ankle inversion in sitting

Sit upright in a chair.
Place your resistance the band around the base of your toes on the exercising foot, and the other end around the leg of the chair or your other foot (wrapped behind you leg that is exercising).
Ensure there is some tension in the band.
Turn your exercising foot inwards against the resistance of the band, keeping your heel in the same position on the floor.
Control the movement as you bring your foot back in to the neutral position.

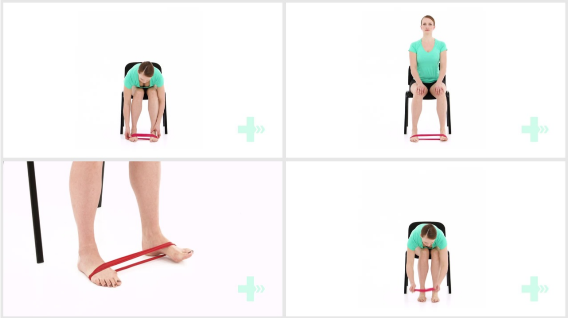

8. Resisted ankle eversion in sitting

Sit upright in a chair with a resistance band tied in a loop.
Place the band around the base of your toes, and move your feet apart to gain some tension in the band.
Keeping your non-exercising foot still, turn your active foot outwards, keeping your heel in the same position on the floor.
Control the movement as you bring your foot back in to the neutral position.

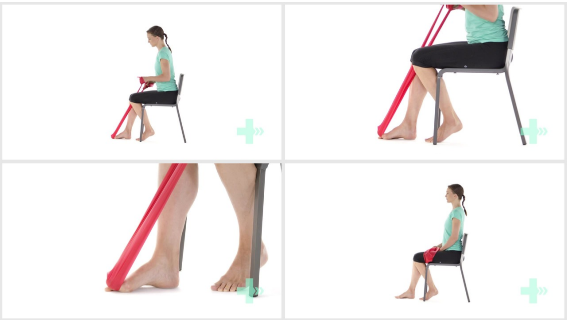

9. Resisted great toe flexion in sitting

Sit in a chair with a resistance band looped around your big toe.
Hold the other end of the band in one hand.
Keeping your other toes firmly on the floor, raise your big toe.
Push your big toe back down towards the floor against the resistance of the band.
Continue to repeat this movement, ensuring your other toes remain on the floor.

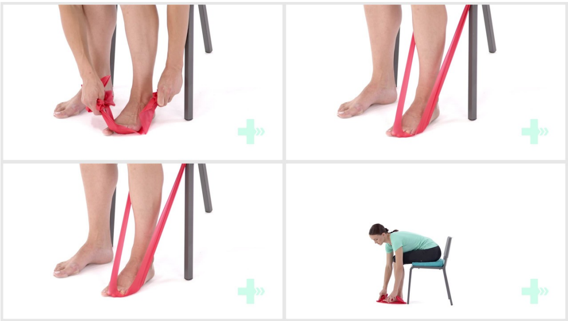

10. Resisted toe flexion

Sit in a chair with a resistance band looped around all of your toes, other than your big toe. Hold the other end of your band in one hand.
Keeping your big toe firmly on the floor, raise your other toes.
Push your toes back down towards the floor against the resistance of the band.
Continue to repeat this movement, ensuring your big toe remains on the floor.

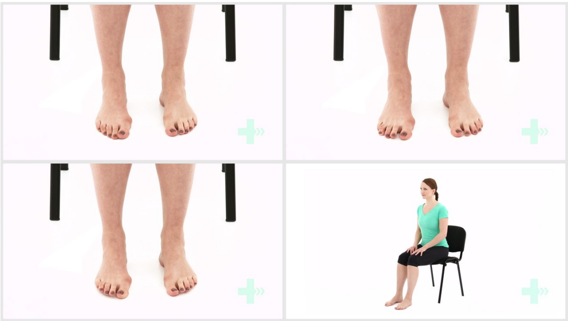

11. Active toe abduction

Sit up straight in a chair with your feet flat on the floor.
Practicing spreading your toes out, particularly your big toe.
Attempt to keep your toes straight and in contact with the floor as you do this.
Alternate repetitions between each foot.

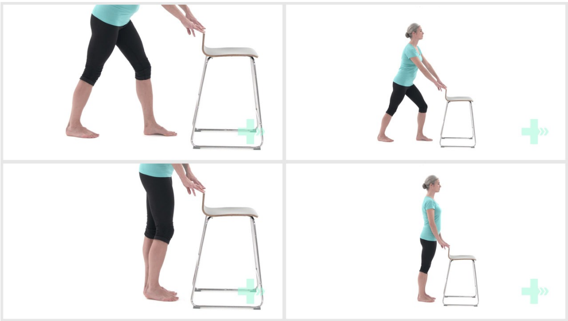

12. Calf stretch standing

In a standing position, hold on to a table or chair for support, or lean against a wall, and step one leg back behind you.
Keep the heel on the floor and the toes pointing forwards.
Bend the front knee, moving your body forwards, until you feel a stretch in the back of the calf.
Make sure your heel does not come off the floor and your back knee does not bend.

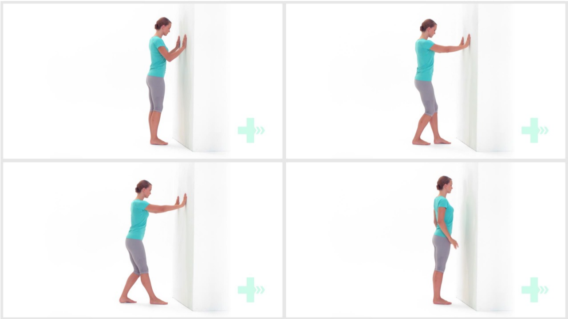

13. Soleus stretching

Start in a standing position, holding on to a wall for support.
Step one leg backwards.
Keeping the toes pointing forwards, bend the front leg, moving your body in this direction.
Hold this position and allow the back knee to soften, your heel may come up a little.
You should feel this stretch lower down in the calf near the tendon

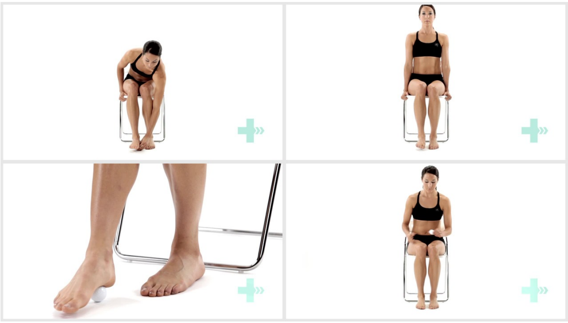

14. Plantar fascia ball roll

Start in a seated position.
Place a small ball underneath the arch of your foot.
Apply pressure down on the ball, and roll the ball from the base of the heel up to the base of the great toe.

Do not do if you find this painful

Week 1 to 4

1. Ankle circles seated

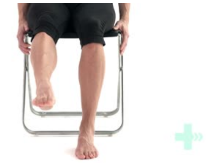

Date:																					
Sets	/3	-	/3	-	/3	-	-	/2	-	/2	-	/2	-	-	/1	-	/1	-	/1	-	-
Reps	/1	-	/1	-	/1	-	-	/1	-	/1	-	/1	-	-	/1	-	/1	-	/1	-	-

2. Toe walking

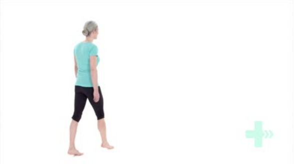

Date:																					
Sets	/2	-	/2	-	/2	-	-	/2	-	/2	-	/2	-	-	/2	-	/2	-	/2	-	-
Reps	/10	-	/10	-	/10	-	-	/12	-	/12	-	/12	-	-	/12	-	/12	-	/14	-	-

3. Walking on heels

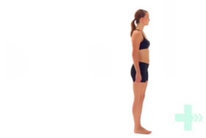

Date:																					
Sets	/2	-	/2	-	/2	-	-	/2	-	/2	-	/2	-	-	/2	-	/2	-	/2	-	-
Reps	/1	-	/1	-	/1	-	-	/1	-	/1	-	/1	-	-	/1	-	/1	-	/1	-	-

4. Alphabet exercise - ankle AROM

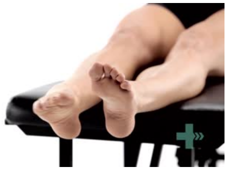

Date:																					
Sets	/1	-	/1	-	/1	-	-	/1	-	/1	-	/1	-	-	/2	-	/2	-	/2	-	-
Reps	/1	-	/1	-	/1	-	-	/1	-	/1	-	/1	-	-	/1	-	/1	-	/1	-	-

5. Metatarsal doming

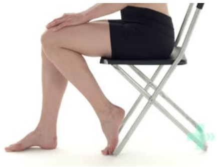

Date:																					
Sets	/5	-	/5	-	/5	-	-	/5	-	/5	-	/5	-	-	/5	-	/5	-	/5	-	-
Reps	/3	-	/3	-	/3	-	-	/3	-	/3	-	/3	-	-	/5	-	/5	-	/5	-	-
Hold	/5	-	/5	-	/5	-	-	/5	-	/5	-	/5	-	-	/5	-	/5	-	/5	-	-

6. Calf raise holding on

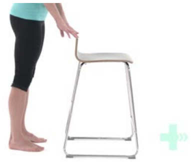

Date:																					
Sets	/1	-	/1	-	/1	-	-	/1	-	/1	-	/1	-	-	/1	-	/1	-	/1	-	-
Reps	/10	-	/10	-	/10	-	-	/10	-	/10	-	/10	-	-	/10	-	/10	-	/10	-	-

7. Resisted ankle inversion in sitting

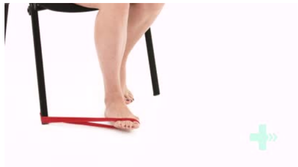

Date:																					
Sets	/1	-	/1	-	/1	-	-	/1	-	/1	-	/1	-	-	/2	-	/2	-	/2	-	-
Reps	/10	-	/10	-	/10	-	-	/10	-	/10	-	/10	-	-	/10	-	/10	-	/10	-	-

8. Resisted ankle eversion in sitting

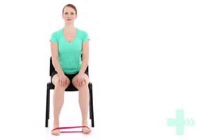

Date:																					
Sets	/1	-	/1	-	/1	-	-	/1	-	/1	-	/1	-	-	/2	-	/2	-	/2	-	-
Reps	/10	-	/10	-	/10	-	-	/10	-	/10	-	/10	-	-	/10	-	/10	-	/10	-	-

9. Resisted great toe flexion in sitting

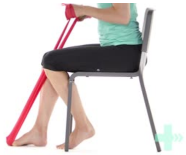

Date:																					
Sets	/2	-	/2	-	/2	-	-	/3	-	/3	-	/3	-	-	/2	-	/2	-	/2	-	-
Reps	/10	-	/10	-	/10	-	-	/10	-	/10	-	/10	-	-	/10	-	/10	-	/10	-	-

10. Resisted toe flexion

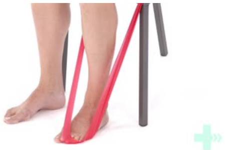

Date:																					
Sets	/10	-	/10	-	/10	-	-	/3	-	/3	-	/3	-	-	/2	-	/2	-	/2	-	-
Reps	/2	-	/2	-	/2	-	-	/10	-	/10	-	/10	-	-	/10	-	/10	-	/10	-	-

11. Active toe abduction

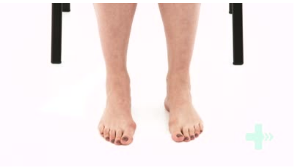

Date:																					
Sets	/1	-	/1	-	/1	-	-	/1	-	/1	-	/1	-	-	/1	-	/1	-	/1	-	-
Reps	/10	-	/10	-	/10	-	-	/10	-	/10	-	/10	-	-	/10	-	/10	-	/10	-	-

12. Calf stretch standing

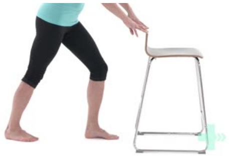

Date:																					
Sets	/2	-	/2	-	/2	-	-	/2	-	/2	-	/2	-	-	/2	-	/2	-	/2	-	-
Reps	/1	-	/1	-	/1	-	-	/1	-	/1	-	/1	-	-	/1	-	/1	-	/1	-	-
Hold	/15	-	/15	-	/15	-	-	/15	-	/15	-	/15	-	-	/15	-	/15	-	/15	-	-

13. Soleus stretching

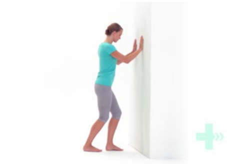

Date:																					
Sets	/1	-	/2	-	/1	-	-	/1	-	/1	-	/1	-	-	/1	-	/1	-	/1	-	-
Reps	/1	-	/1	-	/1	-	-	/1	-	/1	-	/1	-	-	/1	-	/1	-	/1	-	-
Hold	/15	-	/15	-	/15	-	-	/15	-	/15	-	/15	-	-	/15	-	/15	-	/15	-	-

14. Plantar fascia ball roll

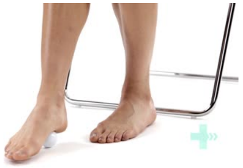

Date:																					
Sets	/2	-	/2	-	/2	-	-	/2	-	/2	-	/2	-	-	/2	-	/2	-	/2	-	-
Reps	/1	-	/1	-	/1	-	-	/1	-	/1	-	/1	-	-	/1	-	/1	-	/1	-	-

1. Ankle circles seated

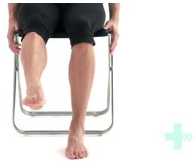

Date:																					
Sets	/1	-	/1	-	/1	-	-	-	-	-	-	-	-	-	-	-	-	-	-	-	-
Reps	/1	-	/1	-	/1	-	-	-	-	-	-	-	-	-	-	-	-	-	-	-	-

2. Toe walking

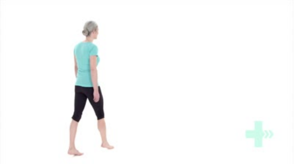

Date:																					
Sets	/2	-	/2	-	/2	-	-	-	-	-	-	-	-	-	-	-	-	-	-	-	-
Reps	/12	-	/12	-	/12	-	-	-	-	-	-	-	-	-	-	-	-	-	-	-	-

3. Walking on heels

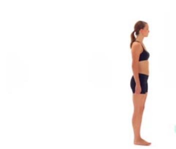

Date:																					
Sets	/2	-	/2	-	/2	-	-	-	-	-	-	-	-	-	-	-	-	-	-	-	-
Reps	/1	-	/1	-	/1	-	-	-	-	-	-	-	-	-	-	-	-	-	-	-	-

4. Alphabet exercise - ankle AROM

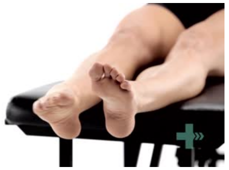

Date:																					
Sets	/2	-	/2	-	/2	-	-	-	-	-	-	-	-	-	-	-	-	-	-	-	-
Reps	/1	-	/1	-	/1	-	-	-	-	-	-	-	-	-	-	-	-	-	-	-	-

5. Metatarsal doming

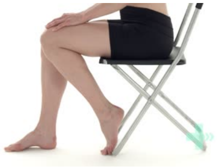

Date:																					
Sets	/5	-	/5	-	/5	-	-	-	-	-	-	-	-	-	-	-	-	-	-	-	-
Reps	/5	-	/5	-	/5	-	-	-	-	-	-	-	-	-	-	-	-	-	-	-	-
Hold	/5	-	/5	-	/5	-	-	-	-	-	-	-	-	-	-	-	-	-	-	-	-

6. Calf raise holding on

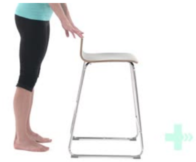

Date:																					
Sets	/1	-	/1	-	/1	-	-	-	-	-	-	-	-	-	-	-	-	-	-	-	-
Reps	/10	-	/10	-	/10	-	-	-	-	-	-	-	-	-	-	-	-	-	-	-	-

7. Resisted ankle inversion in sitting

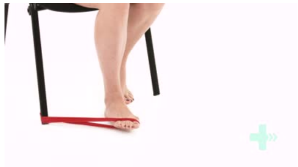

Date:																					
Sets	/2	-	/2	-	/2	-	-	-	-	-	-	-	-	-	-	-	-	-	-	-	-
Reps	/10	-	/10	-	/10	-	-	-	-	-	-	-	-	-	-	-	-	-	-	-	-

8. Resisted ankle eversion in sitting

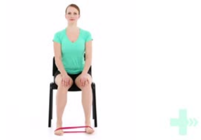

Date:																					
Sets	/2	-	/2	-	/2	-	-	-	-	-	-	-	-	-	-	-	-	-	-	-	-
Reps	/10	-	/10	-	/10	-	-	-	-	-	-	-	-	-	-	-	-	-	-	-	-

9. Resisted great toe flexion in sitting

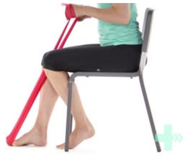

Date:																					
Sets	/3	-	/3	-	/3	-	-	-	-	-	-	-	-	-	-	-	-	-	-	-	-
Reps	/10	-	/10	-	/10	-	-	-	-	-	-	-	-	-	-	-	-	-	-	-	-

10. Resisted toe flexion

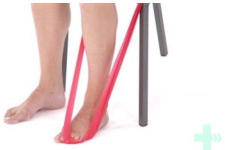

Date:																					
Sets	/3	-	/3	-	/3	-	-	-	-	-	-	-	-	-	-	-	-	-	-	-	-
Reps	/10	-	/10	-	/10	-	-	-	-	-	-	-	-	-	-	-	-	-	-	-	-

11. Active toe abduction

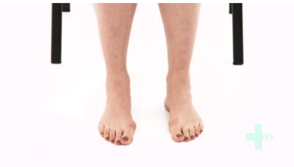

**Date:**																					
Sets	/1	-	/1	-	/1	-	-	-	-	-	-	-	-	-	-	-	-	-	-	-	-
Reps	/10	-	/10	-	/10	-	-	-	-	-	-	-	-	-	-	-	-	-	-	-	-

12. Calf stretch standing

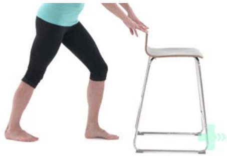

**Date:**																					
Sets	/2	-	/2	-	/2	-	-	-	-	-	-	-	-	-	-	-	-	-	-	-	-
Reps	/1	-	/1	-	/1	-	-	-	-	-	-	-	-	-	-	-	-	-	-	-	-
Hold	/15	-	/15	-	/15	-	-	-	-	-	-	-	-	-	-	-	-	-	-	-	-

13. Soleus stretching

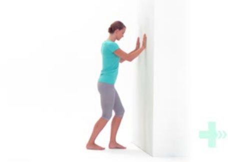

**Date:**																					
Sets	/1	-	/1	-	/1	-	-	-	-	-	-	-	-	-	-	-	-	-	-	-	-
Reps	/1	-	/1	-	/1	-	-	-	-	-	-	-	-	-	-	-	-	-	-	-	-
Hold	/15	-	/15	-	/15	-	-	-	-	-	-	-	-	-	-	-	-	-	-	-	-

14. Plantar fascia ball roll

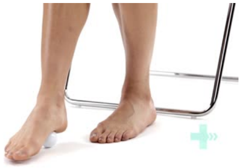

**Date:**																					
Sets	/2	-	/2	-	/2	-	-	-	-	-	-	-	-	-	-	-	-	-	-	-	-
Reps	/1	-	/1	-	/1	-	-	-	-	-	-	-	-	-	-	-	-	-	-	-	-

Description of exercises – see weeks for sets, repetitions and progression

HV Foot strengthening

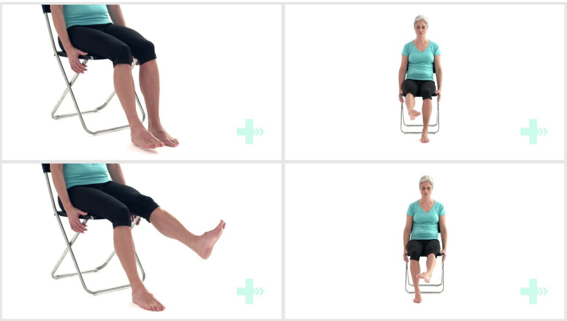

1. Ankle circles seated

The ankle circles help with flexibility of your ankles, this is important for walking and balance.
Sit upright with your back supported before straightening your leg to do the ankle circles.
Lift your leg up and begin making large slow circles with your foot in one direction for the required number of repetitions.
Switch legs and make circles with the other ankle in the same direction.
Switch legs again and reverse the direction of the movement, perform the required number of repetitions firstly with one ankle and then repeat with the other.
Try to stretch your ankle as much as you can as you feel the stretch in your ankle, foot, and in your lower leg.

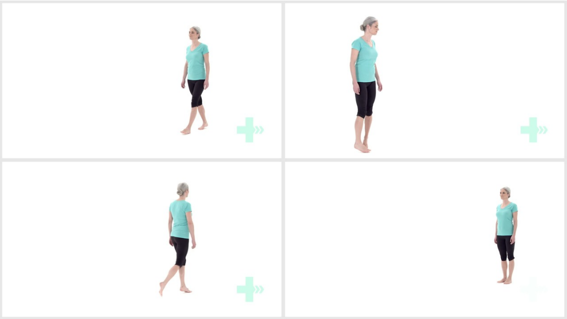

2. Toe walking

Start up on your toes with your heels raised off the floor.
Walk forward with a natural arm swing, keeping your heels off the floor.

Do not do if painful or feeling unbalanced. Alternate between toe and heel walking sets

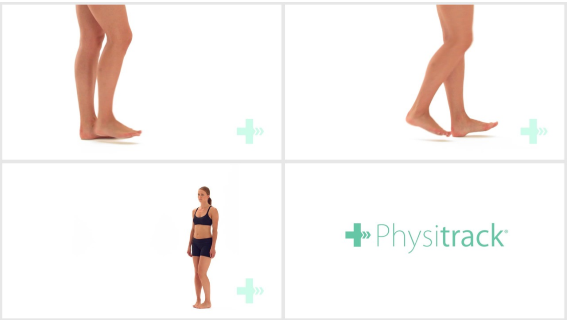

3. Walking on heels

Start from a neutral standing position.
Walk forward balancing only on your heels.

Do not do if you have any heel pain or feel unbalanced

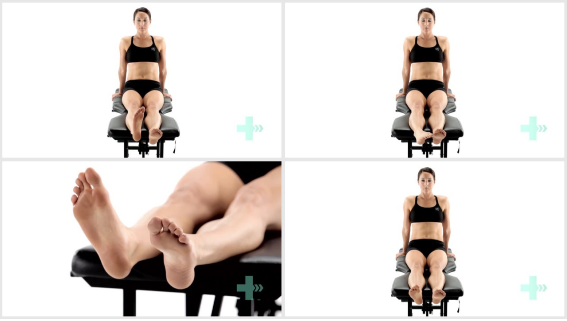

4. Alphabet exercise - ankle AROM

Sit down with your legs extended.
Draw the capital letters of the alphabet with your ankle, accentuating all of the ranges of motion.

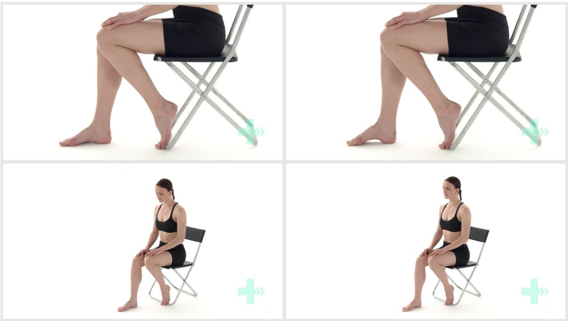

5. Metatarsal doming

Sit up straight in a chair.
Place your foot flat on the floor.
Keeping your toes straight, tighten the muscles through the sole of your foot.
Your arch should raise a little.
Ensure you do not curl your toes under.
You should be able to see your toenails throughout this exercise.
Hold this position.

Progress to standing on one leg. You can use a chair or wall for support.
Hold the position for 5 seconds then relax and repeat for the assigned number of repetitions before switching to the other foot.

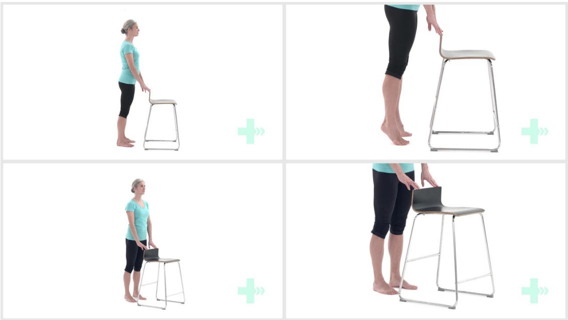

6. Calf raise holding on

Stand with both legs straight and at hip-width apart.
Hold on to a wall or supportive object for balance.
Rise up onto your toes, keeping your legs straight, and control the movement back down to the start position.

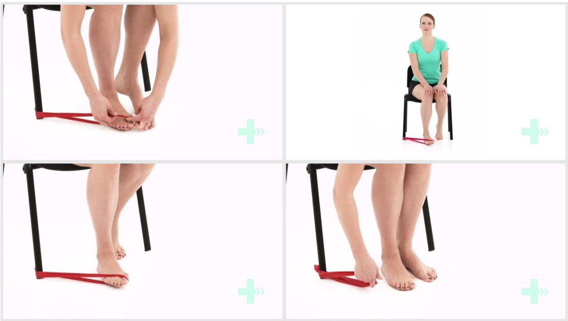

7. Resisted ankle inversion in sitting

Sit upright in a chair.
Place your resistance the band around the base of your toes on the exercising foot, and the other end around the leg of the chair or your other foot (wrapped behind you leg that is exercising).
Ensure there is some tension in the band.
Turn your exercising foot inwards against the resistance of the band, keeping your heel in the same position on the floor.
Control the movement as you bring your foot back in to the neutral position.

If first session of the week felt easy increase the resistance colour of the band from last week

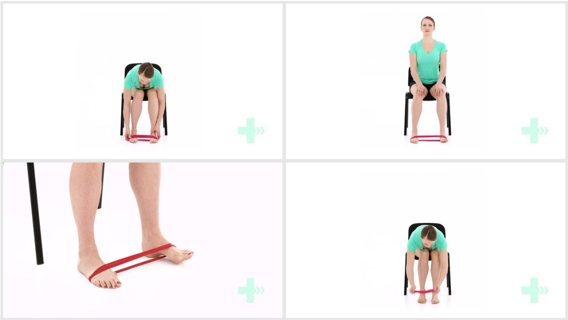

8. Resisted ankle eversion in sitting

Sit upright in a chair with a resistance band tied in a loop.
Place the band around the base of your toes, and move your feet apart to gain some tension in the band.
Keeping your non-exercising foot still, turn your active foot outwards, keeping your heel in the same position on the floor.
Control the movement as you bring your foot back in to the neutral position.

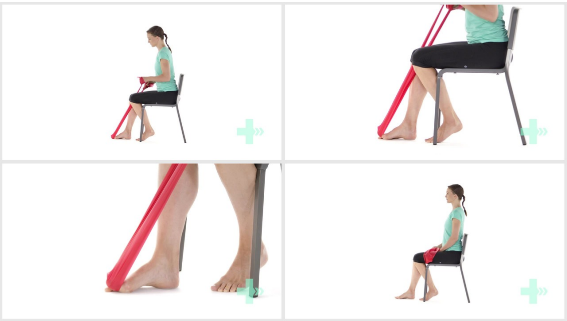

9. Resisted great toe flexion in sitting

Sit in a chair with a resistance band looped around your big toe.
Hold the other end of the band in one hand.
Keeping your other toes firmly on the floor, raise your big toe.
Push your big toe back down towards the floor against the resistance of the band.
Continue to repeat this movement, ensuring your other toes remain on the floor.

Use a resistance band, one heavier than last week

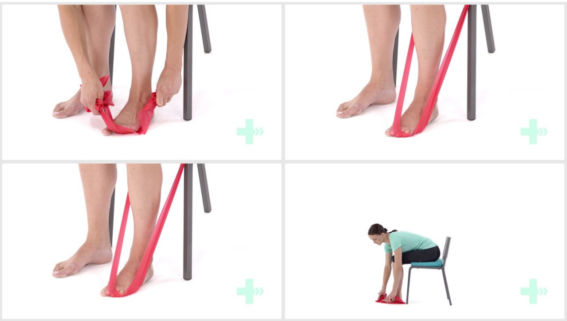

10. Resisted toe flexion

Sit in a chair with a resistance band looped around all of your toes, other than your big toe. Hold the other end of your band in one hand.
Keeping your big toe firmly on the floor, raise your other toes.
Push your toes back down towards the floor against the resistance of the band.
Continue to repeat this movement, ensuring your big toe remains on the floor.

Use a band, one heavier than last week

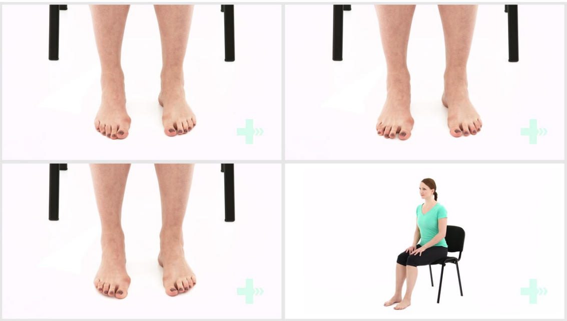

11. Active toe abduction

Sit up straight in a chair with your feet flat on the floor.
Practicing spreading your toes out, particularly your big toe.
Attempt to keep your toes straight and in contact with the floor as you do this.
Alternate repetitions between each foot.

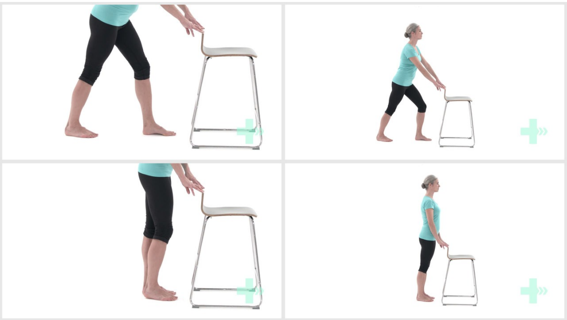

12. Calf stretch standing

In a standing position, hold on to a table or chair for support, or lean against a wall, and step one leg back behind you.
Keep the heel on the floor and the toes pointing forwards.
Bend the front knee, moving your body forwards, until you feel a stretch in the back of the calf.
Make sure your heel does not come off the floor and your back knee does not bend.

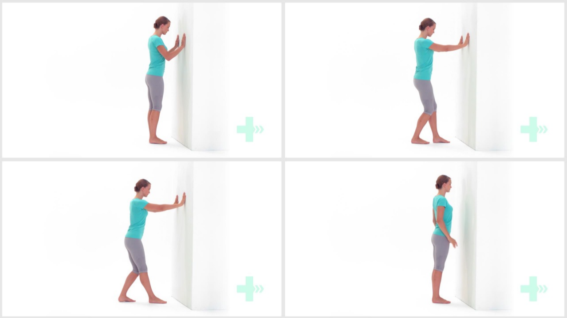

13. Soleus stretching

Start in a standing position, holding on to a wall for support.
Step one leg backwards.
Keeping the toes pointing forwards, bend the front leg, moving your body in this direction.
Hold this position and allow the back knee to soften, your heel may come up a little.
You should feel this stretch lower down in the calf near the tendon

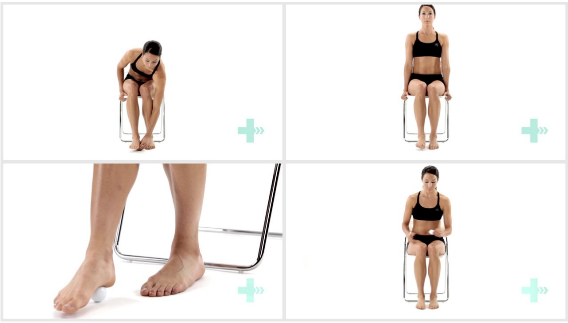

14. Plantar fascia ball roll

Start in a seated position.
Place a small ball underneath the arch of your foot.
Apply pressure down on the ball, and roll the ball from the base of the heel up to the base of the great toe.

Do not do if you find this painful

Week 5 to 8

1. Ankle circles seated

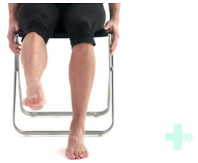

Date:																					
Sets	/1	-	/1	-	/1	-	-	/1	-	/1	-	/1	-	-	/2	-	/1	-	/1	-	-
Reps	/1	-	/1	-	/1	-	-	/1	-	/1	-	/1	-	-	/1	-	/1	-	/1	-	-

2. Toe walking

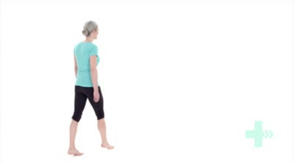

Date:																					
Sets	/2	-	/2	-	/2	-	-	/2	-	/2	-	/2	-	-	/2	-	/2	-	/2	-	-
Reps	/12	-	/12	-	/12	-	-	/12	-	/12	-	/12	-	-	/12	-	/12	-	/12	-	-

3. Walking on heels

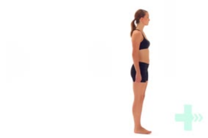

Date:																					
Sets	/2	-	/2	-	/2	-	-	/2	-	/2	-	/2	-	-	/2	-	/2	-	/3	-	-
Reps	/1	-	/1	-	/1	-	-	/1	-	/1	-	/1	-	-	/1	-	/1	-	/1	-	-

4. Alphabet exercise - ankle AROM

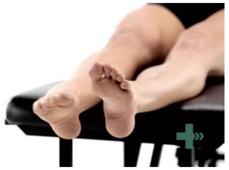

Date:																					
Sets	/2	-	/2	-	/2	-	-	/2	-	/2	-	/2	-	-	/2	-	/2	-	/2	-	-
Reps	/1	-	/1	-	/1	-	-	/1	-	/1	-	/1	-	-	/1	-	/1	-	/1	-	-

5. Metatarsal doming

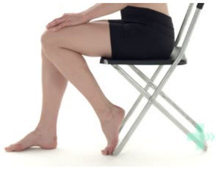

Date:																					
Sets	/3	-	/3	-	/3	-	-	/5	-	/5	-	/5	-	-	/4	-	/4	-	/4	-	-
Reps	/5	-	/5	-	/5	-	-	/3	-	/3	-	/3	-	-	/5	-	/5	-	/5	-	-
Hold	/5	-	/5	-	/5	-	-	/6	-	/6	-	/6	-	-	/6	-	/6	-	/6	-	-

6. Calf raise holding on

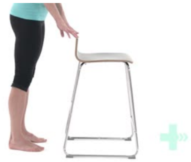

Date:																					
Sets	/2	-	/2	-	/2	-	-	/2	-	/2	-	/2	-	-	/2	-	/2	-	/2	-	-
Reps	/10	-	/10	-	/10	-	-	/10	-	/10	-	/10	-	-	/10	-	/10	-	/10	-	-

7. Resisted ankle inversion in sitting

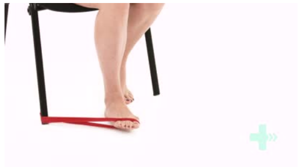

Date:																					
Sets	/2	-	/2	-	/2	-	-	/2	-	/2	-	/2	-	-	/1	-	/1	-	/1	-	-
Reps	/10	-	/10	-	/10	-	-	/10	-	/10	-	/10	-	-	/10	-	/10	-	/10	-	-

8. Resisted ankle eversion in sitting

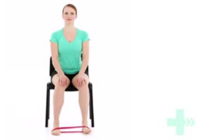

Date:																					
Sets	/2	-	/2	-	/2	-	-	/2	-	/2	-	/2	-	-	/1	-	/1	-	/1	-	-
Reps	/10	-	/10	-	/10	-	-	/10	-	/10	-	/10	-	-	/10	-	/10	-	/10	-	-

9. Resisted great toe flexion in sitting

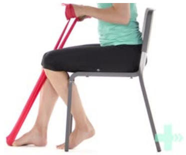

Date:																					
Sets	/2	-	/2	-	/2	-	-	/2	-	/2	-	/2	-	-	/1	-	/1	-	/1	-	-
Reps	/10	-	/10	-	/10	-	-	/10	-	/10	-	/10	-	-	/10	-	/10	-	/10	-	-

10. Resisted toe flexion

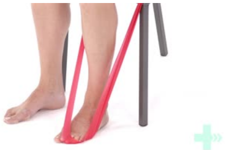

Date:																					
Sets	/2	-	/2	-	/2	-	-	/2	-	/2	-	/2	-	-	/1	-	/1	-	/1	-	-
Reps	/10	-	/10	-	/10	-	-	/10	-	/10	-	/10	-	-	/10	-	/10	-	/10	-	-

11. Active toe abduction

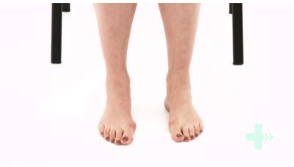

Date:																					
Sets	/1	-	/1	-	/1	-	-	/2	-	/2	-	/2	-	-	/1	-	/1	-	/1	-	-
Reps	/10	-	/10	-	/10	-	-	/10	-	/10	-	/10	-	-	/10	-	/10	-	/10	-	-

12. Calf stretch standing

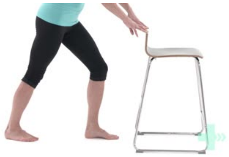

Date:																					
Sets	/2	-	/2	-	/2	-	-	/2	-	/2	-	/2	-	-	/2	-	/2	-	/2	-	-
Reps	/1	-	/1	-	/1	-	-	/1	-	/1	-	/1	-	-	/1	-	/1	-	/1	-	-
Hold	/15	-	/15	-	/15	-	-	/15	-	/15	-	/15	-	-	/15	-	/20	-	/15	-	-

13. Soleus stretching

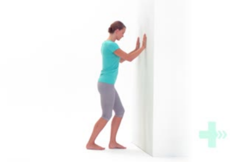

Date:																					
Sets	/1	-	/1	-	/1	-	-	/1	-	/1	-	/1	-	-	/2	-	/1	-	/1	-	-
Reps	/1	-	/1	-	/1	-	-	/1	-	/1	-	/1	-	-	/1	-	/1	-	/1	-	-
Hold	/15	-	/15	-	/15	-	-	/15	-	/15	-	/15	-	-	/15	-	/15	-	/15	-	-

14. Plantar fascia ball roll

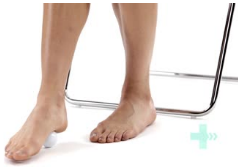

Date:																					
Sets	/2	-	/2	-	/2	-	-	/2	-	/2	-	/2	-	-	/2	-	/2	-	/2	-	-
Reps	/1	-	/1	-	/1	-	-	/1	-	/1	-	/1	-	-	/1	-	/1	-	/1	-	-

1. Ankle circles seated

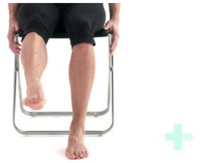

Date:																					
Sets	/2	-	/1	-	/1	-	-	-	-	-	-	-	-	-	-	-	-	-	-	-	-
Reps	/1	-	/1	-	/1	-	-	-	-	-	-	-	-	-	-	-	-	-	-	-	-

2. Toe walking

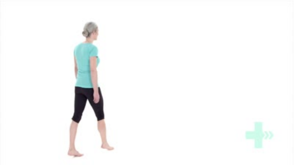

Date:																					
Sets	/2	-	/2	-	/2	-	-	-	-	-	-	-	-	-	-	-	-	-	-	-	-
Reps	/12	-	/12	-	/12	-	-	-	-	-	-	-	-	-	-	-	-	-	-	-	-

3. Walking on heels

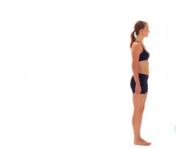

Date:																					
Sets	/2	-	/2	-	/3	-	-	-	-	-	-	-	-	-	-	-	-	-	-	-	-
Reps	/1	-	/1	-	/1	-	-	-	-	-	-	-	-	-	-	-	-	-	-	-	-

4. Alphabet exercise - ankle AROM

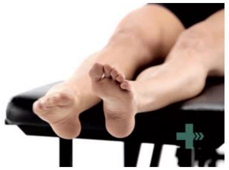

Date:																					
Sets	/2	-	/2	-	/2	-	-	-	-	-	-	-	-	-	-	-	-	-	-	-	-
Reps	/1	-	/1	-	/1	-	-	-	-	-	-	-	-	-	-	-	-	-	-	-	-

5. Metatarsal doming

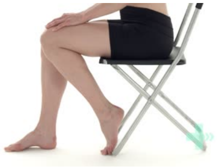

Date:																					
Sets	/4	-	/4	-	/4	-	-	-	-	-	-	-	-	-	-	-	-	-	-	-	-
Reps	/5	-	/5	-	/5	-	-	-	-	-	-	-	-	-	-	-	-	-	-	-	-
Hold	/6	-	/6	-	/6	-	-	-	-	-	-	-	-	-	-	-	-	-	-	-	-

6. Calf raise holding on

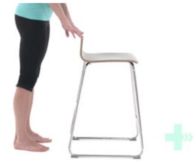

Date:																					
Sets	/2	-	/2	-	/2	-	-	-	-	-	-	-	-	-	-	-	-	-	-	-	-
Reps	/10	-	/10	-	/10	-	-	-	-	-	-	-	-	-	-	-	-	-	-	-	-

7. Resisted ankle inversion in sitting

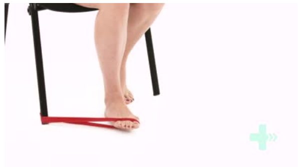

Date:																					
Sets	/1	-	/1	-	/1	-	-	-	-	-	-	-	-	-	-	-	-	-	-	-	-
Reps	/12	-	/12	-	/12	-	-	-	-	-	-	-	-	-	-	-	-	-	-	-	-

8. Resisted ankle eversion in sitting

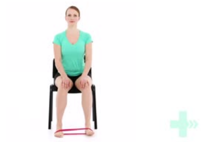

Date:																					
Sets	/1	-	/1	-	/1	-	-	-	-	-	-	-	-	-	-	-	-	-	-	-	-
Reps	/12	-	/12	-	/12	-	-	-	-	-	-	-	-	-	-	-	-	-	-	-	-

9. Resisted great toe flexion in sitting

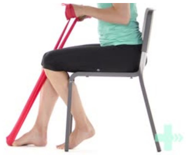

Date:																					
Sets	/2	-	/2	-	/2	-	-	-	-	-	-	-	-	-	-	-	-	-	-	-	-
Reps	/10	-	/10	-	/10	-	-	-	-	-	-	-	-	-	-	-	-	-	-	-	-

10. Resisted toe flexion

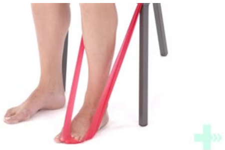

Date:																					
Sets	/2	-	/2	-	/2	-	-	-	-	-	-	-	-	-	-	-	-	-	-	-	-
Reps	/10	-	/10	-	/10	-	-	-	-	-	-	-	-	-	-	-	-	-	-	-	-

11. Active toe abduction

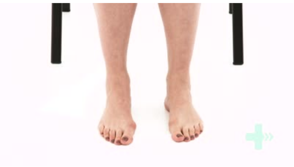

Date:																					
Sets	/2	-	/2	-	/2	-	-	-	-	-	-	-	-	-	-	-	-	-	-	-	-
Reps	/10	-	/10	-	/10	-	-	-	-	-	-	-	-	-	-	-	-	-	-	-	-

12. Calf stretch standing

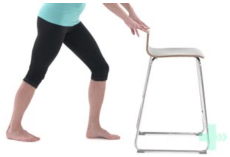

Date:																					
Sets	/2	-	/2	-	/2	-	-	-	-	-	-	-	-	-	-	-	-	-	-	-	-
Reps	/1	-	/1	-	/1	-	-	-	-	-	-	-	-	-	-	-	-	-	-	-	-
Hold	/15	-	/20	-	/15	-	-	-	-	-	-	-	-	-	-	-	-	-	-	-	-

13. Soleus stretching

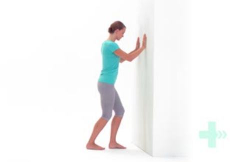

Date:																					
Sets	/2	-	/1	-	/1	-	-	-	-	-	-	-	-	-	-	-	-	-	-	-	-
Reps	/1	-	/1	-	/1	-	-	-	-	-	-	-	-	-	-	-	-	-	-	-	-
Hold	/15	-	/15	-	/15	-	-	-	-	-	-	-	-	-	-	-	-	-	-	-	-

14. Plantar fascia ball roll

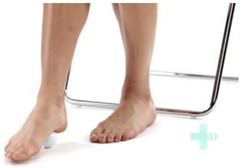

Date:																					
Sets	/2	-	/2	-	/2	-	-	-	-	-	-	-	-	-	-	-	-	-	-	-	-
Reps	/1	-	/1	-	/1	-	-	-	-	-	-	-	-	-	-	-	-	-	-	-	-

Description of exercises – see weeks for sets, repetitions and progression

HV Foot strengthening

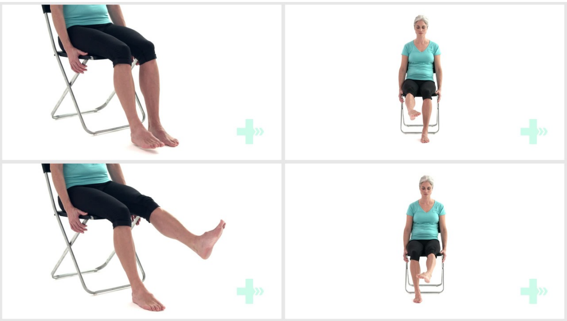

1. Ankle circles seated

The ankle circles help with flexibility of your ankles, this is important for walking and balance.
Sit upright with your back supported before straightening your leg to do the ankle circles.
Lift your leg up and begin making large slow circles with your foot in one direction for the required number of repetitions.
Switch legs and make circles with the other ankle in the same direction.
Switch legs again and reverse the direction of the movement, perform the required number of repetitions firstly with one ankle and then repeat with the other.
Try to stretch your ankle as much as you can as you feel the stretch in your ankle, foot, and in your lower leg.

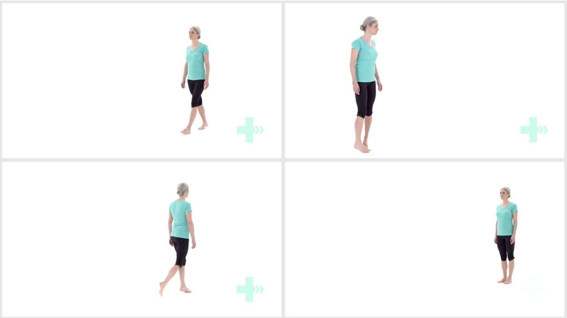

2. Toe walking

Start up on your toes with your heels raised off the floor.
Walk forward with a natural arm swing, keeping your heels off the floor.

Do not do if painful or feeling unbalanced. Alternate between toe and heel walking sets

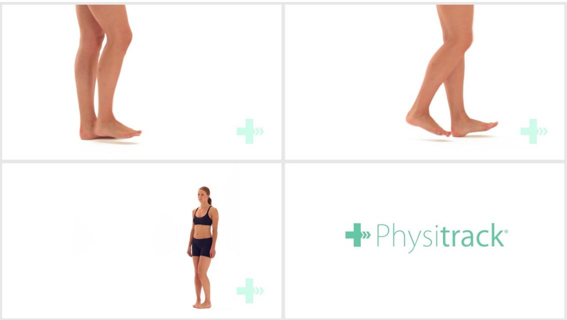

3. Walking on heels

Start from a neutral standing position.
Walk forward balancing only on your heels.

Do not do if you have any heel pain or feel unbalanced

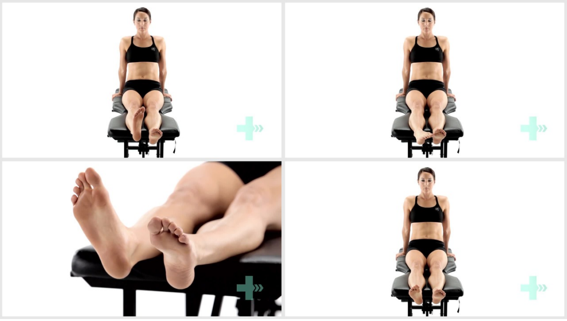

4. Alphabet exercise - ankle AROM

Sit down with your legs extended.
Draw the capital letters of the alphabet with your ankle, accentuating all of the ranges of motion.

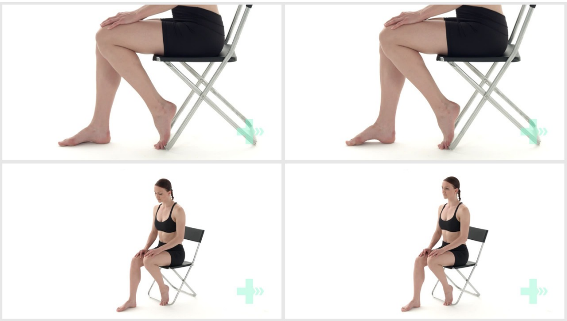

5. Metatarsal doming

Sit up straight in a chair.
Place your foot flat on the floor.
Keeping your toes straight, tighten the muscles through the sole of your foot.
Your arch should raise a little.
Ensure you do not curl your toes under.
You should be able to see your toenails throughout this exercise.
Hold this position.

Progress to standing on one leg. You can use a chair or wall for support.

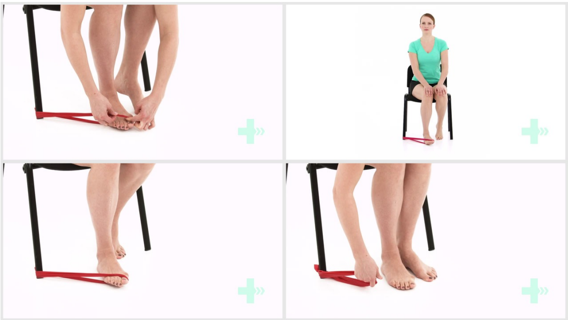

6. Resisted ankle inversion in sitting

Sit upright in a chair.
Place your resistance the band around the base of your toes on the exercising foot, and the other end around the leg of the chair or your other foot (wrapped behind you leg that is exercising).
Ensure there is some tension in the band.
Turn your exercising foot inwards against the resistance of the band, keeping your heel in the same position on the floor.
Control the movement as you bring your foot back in to the neutral position.

If first session of the week felt easy increase the resistance colour of the band from last week

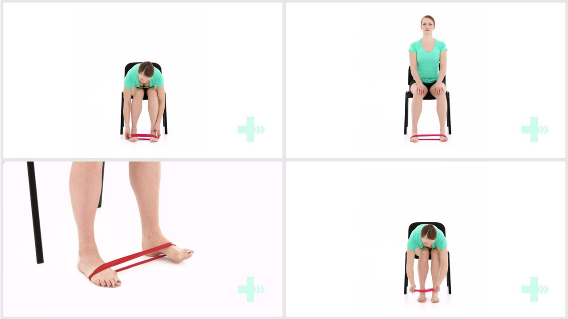

7. Resisted ankle eversion in sitting

Sit upright in a chair with a resistance band tied in a loop.
Place the band around the base of your toes, and move your feet apart to gain some tension in the band.
Keeping your non-exercising foot still, turn your active foot outwards, keeping your heel in the same position on the floor.
Control the movement as you bring your foot back in to the neutral position.

If you didn't increase resistance earlier in the week, Increase the resistance colour of the band from last week

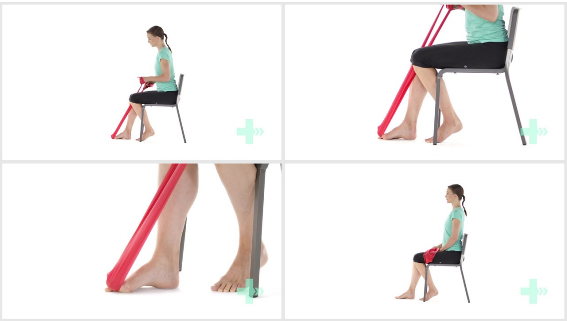

8. Resisted great toe flexion in sitting

Sit in a chair with a resistance band looped around your big toe.
Hold the other end of the band in one hand.
Keeping your other toes firmly on the floor, raise your big toe.
Push your big toe back down towards the floor against the resistance of the band.
Continue to repeat this movement, ensuring your other toes remain on the floor.

Increase strength of band from last week, if you were able to complete without fatigue

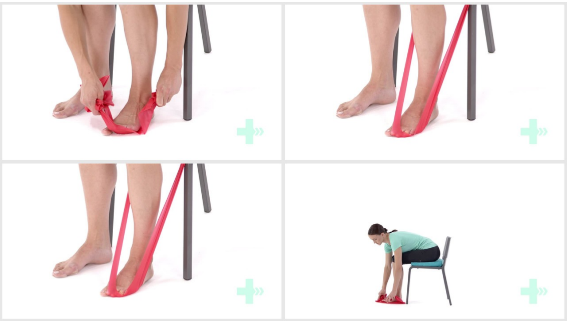

9. Resisted toe flexion

Sit in a chair with a resistance band looped around all of your toes, other than your big toe. Hold the other end of your band in one hand.
Keeping your big toe firmly on the floor, raise your other toes.
Push your toes back down towards the floor against the resistance of the band.
Continue to repeat this movement, ensuring your big toe remains on the floor.

Increase strength of band from last week, if you were able to complete without fatigue

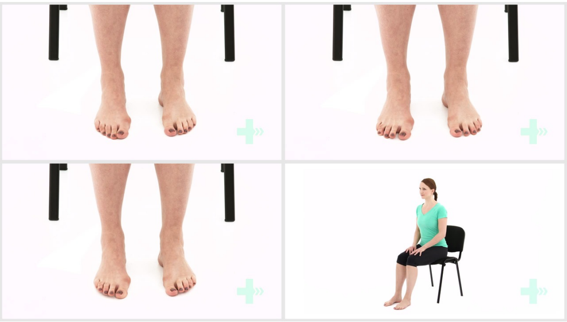

10. Active toe abduction

Sit up straight in a chair with your feet flat on the floor.
Practicing spreading your toes out, particularly your big toe.
Attempt to keep your toes straight and in contact with the floor as you do this.
Alternate repetitions between each foot.

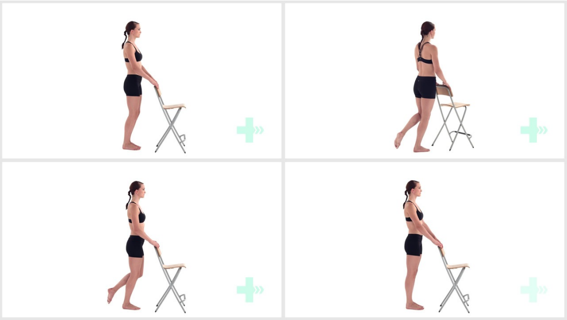

11. Single-leg heel raise

Stand on one leg with your knee slightly bent and your heel flat on the floor. Place a few fingers on the back of a chair, table or wall for balance.
Keeping your knee slightly bent, rise up onto your toes, and control the movement as you lower your heel back down.
Relax and repeat.

If you struggle to complete all the repetitions on one leg, do as many as you can before finishing off the remaining repetitions on both legs, then switch to the opposite leg.

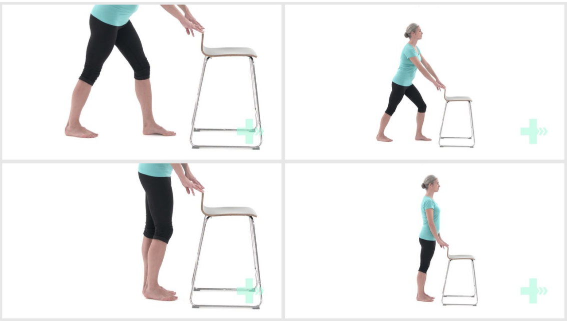

12. Calf stretch standing

In a standing position, hold on to a table or chair for support, or lean against a wall, and step one leg back behind you.
Keep the heel on the floor and the toes pointing forwards.
Bend the front knee, moving your body forwards, until you feel a stretch in the back of the calf.
Make sure your heel does not come off the floor and your back knee does not bend.

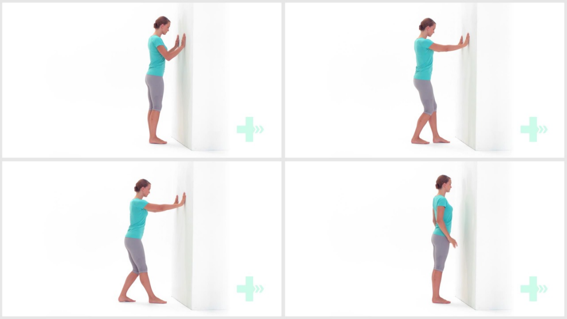

13. Soleus stretching

Start in a standing position, holding on to a wall for support.
Step one leg backwards.
Keeping the toes pointing forwards, bend the front leg, moving your body in this direction.
Hold this position and allow the back knee to soften, your heel may come up a little.
You should feel this stretch lower down in the calf near the tendon

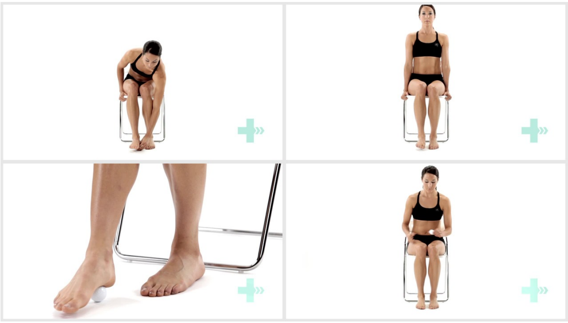

14. Plantar fascia ball roll

Start in a seated position.
Place a small ball underneath the arch of your foot.
Apply pressure down on the ball, and roll the ball from the base of the heel up to the base of the great toe.

Do not do if you find this painful

Week 9 to 12

1. Ankle circles seated

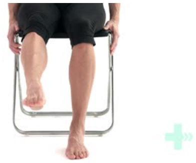

Date:																					
Sets	/2	-	/1	-	/1	-	-	/2	-	/1	-	/1	-	-	/2	-	/1	-	/1	-	-
Reps	/1	-	/1	-	/1	-	-	/1	-	/1	-	/1	-	-	/1	-	/1	-	/1	-	-

2. Toe walking

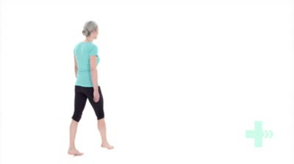

Date:																					
Sets	/2	-	/2	-	/2	-	-	/2	-	/2	-	/2	-	-	/2	-	/2	-	/2	-	-
Reps	/12	-	/12	-	/12	-	-	/12	-	/12	-	/12	-	-	/12	-	/12	-	/12	-	-

3. Walking on heels

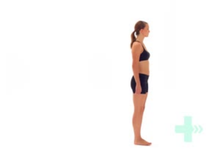

Date:																					
Sets	/2	-	/2	-	/3	-	-	/2	-	/2	-	/3	-	-	/2	-	/2	-	/3	-	-
Reps	/1	-	/1	-	/1	-	-	/1	-	/1	-	/1	-	-	/1	-	/1	-	/1	-	-

4. Alphabet exercise - ankle AROM

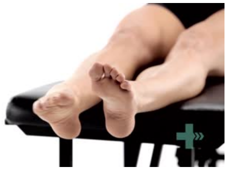

Date:																					
Sets	/2	-	/2	-	/2	-	-	/2	-	/2	-	/2	-	-	/2	-	/2	-	/2	-	-
Reps	/1	-	/1	-	/1	-	-	/1	-	/1	-	/1	-	-	/1	-	/1	-	/1	-	-

5. Metatarsal doming

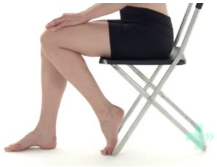

Date:																					
Sets	/5	-	/5	-	/5	-	-	/5	-	/5	-	/5	-	-	/5	-	/5	-	/5	-	-
Reps	/5	-	/5	-	/5	-	-	/5	-	/5	-	/5	-	-	/5	-	/5	-	/5	-	-
Hold	/5	-	/5	-	/5	-	-	/6	-	/6	-	/6	-	-	/8	-	/8	-	/8	-	-

6. Resisted ankle inversion in sitting

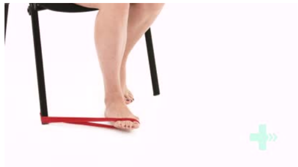

Date:																					
Sets	/1	-	/1	-	/1	-	-	/2	-	/2	-	/2	-	-	/2	-	/2	-	/2	-	-
Reps	/15	-	/15	-	/15	-	-	/10	-	/10	-	/10	-	-	/12	-	/12	-	/12	-	-

7. Resisted ankle eversion in sitting

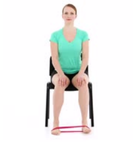

Date:																					
Sets	/1	-	/1	-	/1	-	-	/2	-	/2	-	/2	-	-	/2	-	/2	-	/2	-	-
Reps	/15	-	/15	-	/15	-	-	/10	-	/10	-	/10	-	-	/12	-	/12	-	/12	-	-

8. Resisted great toe flexion in sitting

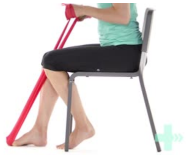

Date:																					
Sets	/2	-	/2	-	/2	-	-	/2	-	/2	-	/2	-	-	/2	-	/2	-	/2	-	-
Reps	/10	-	/10	-	/10	-	-	/10	-	/10	-	/10	-	-	/15	-	/15	-	/15	-	-

9. Resisted toe flexion

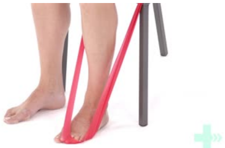

Date:																					
Sets	/2	-	/2	-	/2	-	-	/2	-	/2	-	/2	-	-	/2	-	/2	-	/2	-	-
Reps	/10	-	/10	-	/10	-	-	/10	-	/10	-	/10	-	-	/15	-	/15	-	/15	-	-

10. Active toe abduction

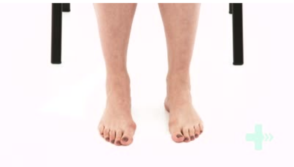

Date:																					
Sets	/2	-	/2	-	/2	-	-	/2	-	/2	-	/2	-	-	/2	-	/2	-	/2	-	-
Reps	/10	-	/10	-	/10	-	-	/10	-	/10	-	/10	-	-	/10	-	/10	-	/10	-	-

11. Single-leg soleus heel raise

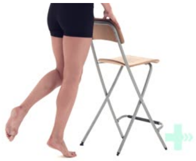

Date:																					
Sets	/1	-	/1	-	/1	-	-	/1	-	/1	-	/1	-	-	/1	-	/1	-	/1	-	-
Reps	/10	-	/10	-	/10	-	-	/10	-	/10	-	/10	-	-	/10	-	/10	-	/10	-	-

12. Calf stretch standing

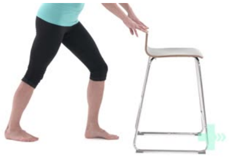

Date:																					
Sets	/2	-	/2	-	/2	-	-	/2	-	/2	-	/2	-	-	/2	-	/2	-	/2	-	-
Reps	/1	-	/1	-	/1	-	-	/1	-	/1	-	/1	-	-	/1	-	/1	-	/1	-	-
Hold	/15	-	/20	-	/15	-	-	/15	-	/20	-	/15	-	-	/15	-	/20	-	/15	-	-

13. Soleus stretching

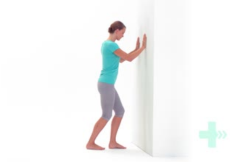

Date:																					
Sets	/2	-	/1	-	/1	-	-	/2	-	/1	-	/1	-	-	/2	-	/1	-	/1	-	-
Reps	/1	-	/1	-	/1	-	-	/1	-	/1	-	/1	-	-	/1	-	/1	-	/1	-	-
Hold	/15	-	/15	-	/15	-	-	/15	-	/15	-	/15	-	-	/15	-	/15	-	/15	-	-

14. Plantar fascia ball roll

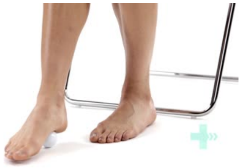

Date:																					
Sets	/2	-	/2	-	/2	-	-	/2	-	/2	-	/2	-	-	/2	-	/2	-	/2	-	-
Reps	/1	-	/1	-	/1	-	-	/1	-	/1	-	/1	-	-	/1	-	/1	-	/1	-	-

1. Ankle circles seated

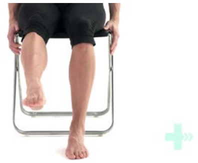

Date:																					
Sets	/2	-	/1	-	/1	-	-	-	-	-	-	-	-	-	-	-	-	-	-	-	-
Reps	/1	-	/1	-	/1	-	-	-	-	-	-	-	-	-	-	-	-	-	-	-	-

2. Toe walking

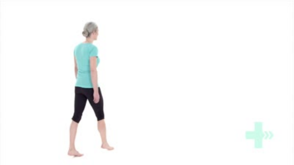

Date:																					
Sets	/2	-	/2	-	/2	-	-	-	-	-	-	-	-	-	-	-	-	-	-	-	-
Reps	/12	-	/12	-	/12	-	-	-	-	-	-	-	-	-	-	-	-	-	-	-	-

3. Walking on heels

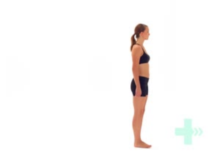

Date:																					
Sets	/2	-	/2	-	/3	-	-	-	-	-	-	-	-	-	-	-	-	-	-	-	-
Reps	/1	-	/1	-	/1	-	-	-	-	-	-	-	-	-	-	-	-	-	-	-	-

4. Alphabet exercise - ankle AROM

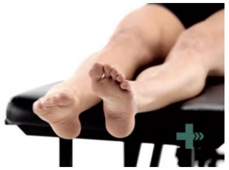

Date:																					
Sets	/2	-	/2	-	/2	-	-	-	-	-	-	-	-	-	-	-	-	-	-	-	-
Reps	/1	-	/1	-	/1	-	-	-	-	-	-	-	-	-	-	-	-	-	-	-	-

5. Metatarsal doming

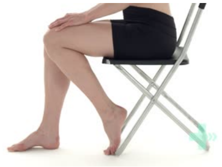

Date:																					
Sets	/5	-	/5	-	/5	-	-	-	-	-	-	-	-	-	-	-	-	-	-	-	-
Reps	/5	-	/5	-	/5	-	-	-	-	-	-	-	-	-	-	-	-	-	-	-	-
Hold	/10	-	/10	-	/10	-	-	-	-	-	-	-	-	-	-	-	-	-	-	-	-

6. Resisted ankle inversion in sitting

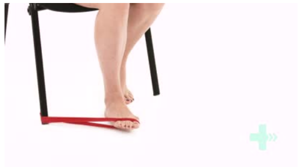

Date:																					
Sets	/2	-	/2	-	/2	-	-	-	-	-	-	-	-	-	-	-	-	-	-	-	-
Reps	/15	-	/15	-	/15	-	-	-	-	-	-	-	-	-	-	-	-	-	-	-	-

7. Resisted ankle eversion in sitting

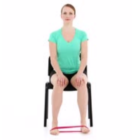

Date:																					
Sets	/2	-	/2	-	/2	-	-	-	-	-	-	-	-	-	-	-	-	-	-	-	-
Reps	/15	-	/15	-	/15	-	-	-	-	-	-	-	-	-	-	-	-	-	-	-	-

8. Resisted great toe flexion in sitting

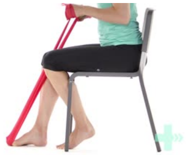

Date:																					
Sets	/2	-	/2	-	/2	-	-	-	-	-	-	-	-	-	-	-	-	-	-	-	-
Reps	/15	-	/15	-	/15	-	-	-	-	-	-	-	-	-	-	-	-	-	-	-	-

9. Resisted toe flexion

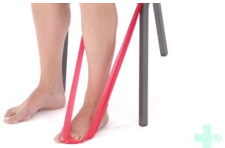

Date:																					
Sets	/2	-	/2	-	/2	-	-	-	-	-	-	-	-	-	-	-	-	-	-	-	-
Reps	/15	-	/15	-	/15	-	-	-	-	-	-	-	-	-	-	-	-	-	-	-	-

10. Active toe abduction

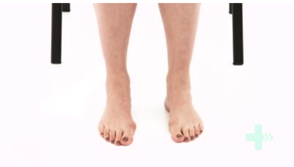

Date:																					
Sets	/2	-	/2	-	/2	-	-	-	-	-	-	-	-	-	-	-	-	-	-	-	-
Reps	/10	-	/10	-	/10	-	-	-	-	-	-	-	-	-	-	-	-	-	-	-	-

11. Single-leg soleus heel raise

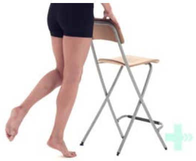

Date:																					
Sets	/1	-	/1	-	/1	-	-	-	-	-	-	-	-	-	-	-	-	-	-	-	-
Reps	/10	-	/10	-	/10	-	-	-	-	-	-	-	-	-	-	-	-	-	-	-	-

12. Calf stretch standing

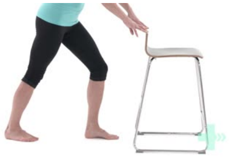

Date:																					
Sets	/2	-	/2	-	/2	-	-	-	-	-	-	-	-	-	-	-	-	-	-	-	-
Reps	/1	-	/1	-	/1	-	-	-	-	-	-	-	-	-	-	-	-	-	-	-	-
Hold	/15	-	/20	-	/15	-	-	-	-	-	-	-	-	-	-	-	-	-	-	-	-

13. Soleus stretching

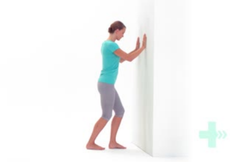

Date:																					
Sets	/2	-	/1	-	/1	-	-	-	-	-	-	-	-	-	-	-	-	-	-	-	-
Reps	/1	-	/1	-	/1	-	-	-	-	-	-	-	-	-	-	-	-	-	-	-	-
Hold	/15	-	/15	-	/15	-	-	-	-	-	-	-	-	-	-	-	-	-	-	-	-

14. Plantar fascia ball roll

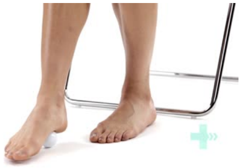

Date:																					
Sets	/2	-	/2	-	/2	-	-	-	-	-	-	-	-	-	-	-	-	-	-	-	-
Reps	/1	-	/1	-	/1	-	-	-	-	-	-	-	-	-	-	-	-	-	-	-	-
